# Effects of narrative‐based interventions on self‐efficacy and self‐management in chronic disease: A systematic review and meta‐analysis

**DOI:** 10.1111/aphw.70127

**Published:** 2026-02-17

**Authors:** Zhuangyan Yao, Edmond Pui Hang Choi, Do Do Wai Nei Chow, Hong Chen, Lizhen Wang, Mu‐Hsing Ho

**Affiliations:** ^1^ Affiliated Hospital of Guangdong Medical University Zhanjiang China; ^2^ School of Nursing Li Ka Shing Faculty of Medicine The University of Hong Kong Hong Kong SAR China

**Keywords:** chronic disease, narrative‐based intervention, randomized controlled trial, self‐efficacy, self‐management

## Abstract

This systematic review and meta‐analysis aimed to evaluate the impact of narrative interventions on self‐efficacy and self‐management behaviors in patients with chronic diseases. We systematically searched EMBASE, the Cochrane Library, CINAHL, PsycINFO, China National Knowledge Infrastructure (CNKI), and PubMed from conception to 2025, supplemented by manual searches of reference lists. Randomized controlled trials (RCTs) assessing the effects of narrative interventions on self‐efficacy or self‐management in adults with chronic diseases were included. Data were pooled using random‐effects meta‐analysis. A total of 34 RCTs involving 4584 participants were included. The meta‐analysis showed that narrative‐based interventions significantly enhanced patients' self‐efficacy (22 studies; *SMD* = 0.66, 95% CI: 0.35–0.96, *p* < .001; *I* ^*2*^ = 59.6%) and self‐management behaviors (12 studies; *SMD* = 1.73, 95% CI: 1.03–2.42, *p* < .001; *I* ^*2*^ = 85.1%). Meta‐regression indicated a positive regulatory trend of national income level on self‐efficacy (*p* = .055), although the effect was not statistically significant, and no significant moderating effect was observed for self‐management. Other variables—including age, disease type, intervention type, duration, and publication period—showed no significant moderating impact on either outcome (*p* > .05). Subgroup analysis further revealed that the improvement in self‐efficacy was significantly greater in upper‐ to middle‐income countries (*SMD* = 1.04) than in high‐income countries (*SMD* = 0.39; subgroup difference *p* = .019), and the effect was significant among patients with heart disease (*SMD* = 1.16), cancer (*SMD* = 0.98) and stroke (*SMD* = 0.71). For self‐management, the effect varied significantly across subgroups defined by intervention type, duration, publication period and disease type, with the largest effects observed among patients with neurological diseases (*SMD* = 3.16) and cancer (*SMD* = 2.73). The results of this systematic review and meta‐analysis suggest that narrative‐based interventions may improve self‐efficacy and self‐management behaviors among patients with chronic diseases. However, considerable heterogeneity remains, and further high‐quality studies are needed to confirm these findings.

## INTRODUCTION

Chronic diseases are characterized by a prolonged course and slow progression, and their onset is closely linked to the complex interplay of factors such as genetics, physiology, behavior, and environment (World Health Organization, 2024). Unlike acute conditions, managing chronic diseases not only relies on medical interventions and symptom control but also requires the maintenance of long‐term healthy behaviors and adaptive adjustments in psychological and social functioning (Duda‐Sikuła & Kurpas, 2024). With the aging global population and significant lifestyle changes, the prevalence of chronic diseases continues to rise, posing a major challenge to global public health (Anderson & Durstine, 2019; Rudnicka et al., 2020). According to the latest report from the World Health Organization (2024), non‐communicable diseases account for approximately 41 million deaths annually, representing 74% of total global mortality, with leading causes including cardiovascular diseases, cancer, chronic respiratory diseases, and diabetes. These conditions persist in imposing physical strain and restricting functional capabilities on patients, while also profoundly impacting their psychological well‐being and social interactions. As a result, they create significant challenges for individuals, families, and society at large (Christiansen et al., 2021). Furthermore, the rising incidence of chronic illnesses is placing extraordinary strain on global health‐care infrastructures and economic systems (World Health Organization, 2024). In response to this global challenge, the WHO launched the Global Action Plan for the Prevention and Control of Non‐communicable Diseases (2013–2020) in 2013, which emphasizes the reduction of morbidity and mortality from chronic diseases through multisectoral collaboration, health promotion, and disease management (World Health Organization, 2013). However, despite various measures taken at the policy level, the prevention and control of chronic diseases remains unsatisfactory, especially in low‐ and middle‐income countries (Mwangi et al., 2021).

Enhancing patients' self‐management abilities is widely recognized as a core strategy for addressing the challenges associated with chronic disease management (Lorig & Holman, 2003; Richardson et al., 2014; Timmermans et al., 2023). Effective self‐management involves a range of behaviors, including adhering to medical advice, maintaining a balanced diet, engaging in regular physical activity, and continuously monitoring one's health status (Iovino et al., 2024; Kim et al., 2021). However, despite its indispensable role in improving clinical outcomes, patients often encounter considerable barriers when attempting to sustain these behaviors over the long term. These obstacles stem from multiple sources, such as perceived difficulties, fluctuating emotions and psychological states, and the practical challenges of integrating complex medical advice into daily life (Yıldırım, 2020). Research has shown that an individual's belief in their ability to carry out and adhere to self‐management behaviors—known as self‐efficacy—plays a critical role in overcoming these challenges (Chan, 2021). According to Bandura's (1977) Social Cognitive Theory, self‐efficacy influences not only motivation and behavioral choices but also the degree of persistence and emotional responses when facing obstacles. Importantly, self‐efficacy and self‐management behavior have a mutually reinforcing relationship. A substantial body of empirical evidence indicates that self‐efficacy is a key psychological antecedent that drives and sustains effective self‐management behaviors, such as medication adherence, symptom monitoring, and lifestyle modification (Chen et al., 2023; González‐Conde et al., 2019; Montalbano et al., 2023). Meanwhile, successful self‐management experiences further strengthen patients' self‐efficacy, creating a positive feedback loop (Huang, Li, et al., 2024).

However, this virtuous cycle is often difficult to initiate and sustain among adolescents. Gauci et al. (2021) reported in a systematic review of face‐to‐face, skills‐based self‐management programs (including educational sessions, behavioral skills training, and problem‐solving approaches) for adolescents with chronic diseases that, although such interventions may yield short‐term improvements in treatment adherence, they generally fail to produce clinically meaningful or durable long‐term effects. The review found that while some included studies demonstrated immediate or short‐term positive changes following the intervention, none provided evidence of sustained benefits. Over time, patients' adherence tended to decline or revert to baseline levels, and improvements in self‐efficacy were similarly difficult to maintain. This widespread limitation has prompted researchers to engage in deeper reflection. Traditional intervention strategies that focus primarily on knowledge dissemination and behavioral guidance may have inherent shortcomings in effectively and sustainably enhancing self‐efficacy (Farley, 2020). These approaches tend to rely on one‐way information delivery and often overlook patients' individual experiences and emotional needs, thereby failing to provide the contextual resonance and deeper psychological support required to genuinely motivate behavioral change (Devan et al., 2018).

Further evidence suggests that many existing self‐management programs do not systematically incorporate emotion regulation or physiological arousal management strategies—both of which play key roles in shaping self‐efficacy—ultimately limiting their clinical applicability and long‐term impact (Benzo, 2024; Boger et al., 2015; Jiang et al., 2019). As a result, identifying new intervention strategies that can more deeply and sustainably enhance self‐efficacy has become a critical and urgent priority in improving chronic disease self‐management. Against this backdrop, narrative‐based intervention—a patient‐centered psychological and behavioral approach—has attracted growing attention in recent years (Yang et al., 2020). The core of narrative‐based intervention lies in guiding patients to express, listen to, and reconstruct their illness‐related experiences in a structured manner, transforming internal, often fragmented or distressing experiences into coherent, shareable, and meaningful life stories (Yang et al., 2020).

The application forms of narrative‐based intervention are diverse. Common forms include digital storytelling, which presents first‐person accounts of patients or fictional characters through videos, multimedia, or animations to enhance emotional engagement and vicarious experience (Moreau et al., 2018); information‐based narrative messages, which embed health knowledge, behavioral recommendations, or treatment information within a storyline to improve comprehension and acceptance (Cates et al., 2015); and interpersonal or oral narratives, which facilitate meaning‐making, social support, and psychological adjustment through the telling and listening of illness experiences in individual interviews or group discussions (Roikjær et al., 2022). Additional forms include expressive or narrative writing and testimonial narratives, in which patients share their personal experiences (Roikjær et al., 2022).

The effectiveness of narrative‐based intervention has been preliminarily demonstrated across a variety of chronic disease contexts. For example, this approach has been successfully applied in diabetes management, stroke rehabilitation, and cancer care, where it has been shown to reduce anxiety and depression and enhance patients' adherence to treatment plans (Andreae et al., 2021; Appalasamy, Quek, et al., 2020; Crogan et al., 2008). In chronic pain management, narrative intervention has been found to strengthen patients' confidence in managing their symptoms, facilitate identification with peers' experiences, and thereby improve coping strategies and overall quality of life (Lopez‐Olivo, Des Bordes, et al., 2021; Perrier & Martin Ginis, 2017). In adolescent cancer patients, digital narrative creation enables the sharing of personal experiences, promotes trauma healing, and helps integrate past adversity with current life. For patients with dementia, narrative intervention can meet both physical and psychological needs, improve daily functioning, and delay cognitive decline (Laing et al., 2017).

From a theoretical perspective, social cognitive theory (Bandura, 1989) provides the core foundation for narrative‐based interventions, emphasizing that through vicarious experiences and observational learning, individuals can enhance self‐efficacy and thereby strengthen their capacity to manage chronic diseases (Shaffer et al., 2018). The elaboration likelihood model (Petty & Cacioppo, 1986) suggests that narratives influence individuals through two pathways: the central route, which stimulates deep cognitive processing, and the peripheral route, which triggers emotional resonance, thereby jointly promoting attitude change. The empathy elicited by narratives also enhances audience identification and emotional engagement with the story (Gesser‐Edelsburg, 2021; Hinyard & Kreuter, 2006). Compared with traditional didactic approaches, narrative‐based interventions have shown significant advantages in improving health‐related knowledge, attitudes, and behavioral intentions (Bell et al., 2021; Murphy et al., 2013). This advantage arises from narratives' ability to reduce psychological resistance to health information and enhance comprehension and memory retention (Bell et al., 2021; Falzon et al., 2015). Belief system theory (Rokeach, 1968) further explains the capacity of narrative‐based interventions to reshape individuals' core values and beliefs; when narrative content aligns with or constructively challenges a person's worldview, it can facilitate lasting behavioral change (Appalasamy, Quek, et al., 2020). The narrative immersion model provides additional support, suggesting that deep immersion and identification with a story can promote belief adjustment and emotional responses, thereby effectively predicting changes in health behaviors (Lee et al., 2015; Shaffer et al., 2018). Together, these theories form an integrated framework that systematically clarifies how narrative‐based interventions promote health behavior change through the synergistic effects of cognition, emotion, and belief.

Nevertheless, studies in this area exhibit significant variation in both theoretical foundations and methodological approaches. Numerous investigations are hindered by the absence of a standardized framework for categorizing narrative forms and explaining their underlying mechanisms, which limits their practical application in clinical settings and complicates the integration of research findings (Gucciardi et al., 2016; Winterbottom et al., 2008). Therefore, this study aims to conduct a systematic review and meta‐analysis to comprehensively evaluate the impact of narrative intervention on the self‐efficacy and self‐management behaviors of patients with chronic diseases. It also aims to explore potential moderating factors, including intervention type, disease type, intervention duration, publication time, and self‐efficacy type to identify the sources of heterogeneity. The research results are expected to provide high‐quality evidence for the effectiveness of this intervention measure, fill the existing research gaps, and provide guidance for developing more patient‐centered and evidence‐based chronic disease management methods.

## METHODOLOGY

This systematic review protocol has been registered in the International Prospective Systematic Review Register (PROSPERO). Systematic reviews and meta‐analyses complied with the PRISMA guidelines and the Cochrane Handbook of Systematic Reviews (Higgins et al., 2022).

### Inclusion and exclusion criteria

Based on the population, intervention, comparison, and outcome criteria, studies that met the following criteria were included: (1) Participants were diagnosed with chronic diseases as defined by the world health organization (WHO), including cardiovascular disease, diabetes, or cancer; (2) randomized control trails (RCTs) with narrative therapy, storytelling, narrative information, narrative diary, and other interventions related to the narrative; (3) the control group received usual care or placebo intervention; (4) self‐efficacy (the individual's confidence in their ability to take specific actions in managing chronic diseases [measured through validated self‐reporting scales] or self‐management behavior; (5) unlimited publication time, and only articles published in peer‐reviewed journals were considered.

Exclusion criteria: (1) Studies with no control group, or non‐RCTs (e.g., observational studies, conference papers, thesis, etc.); (2) studies that did not report self‐efficacy or self‐management data; and (3) studies that have not been published in either Chinese or English were excluded

### Search strategy

A comprehensive search of six databases, including EMBASE, Cochrane Controlled Trials Centre, CINAHL, PsycINFO, CNKI, and PubMed, was conducted. A librarian was consulted to develop the search strategy. Search strategies were shown in Supplementary Table S1. Medical subject headings and free text keywords related to narrative‐based interventions were used from inception through August 2025. The reviewers also manually searched the reference lists of previously reviewed papers and included studies. The studies included in this review were RCTs of any design, published in either English or Chinese.

### Study selection

The screening process in this study was divided into several steps. First, the reviewer conducted an initial screening of titles and abstracts based on the research question and the inclusion and exclusion criteria of this review. Subsequently, two reviewers further screened the abstracts after the initial screening to identify the literature that required full reading. If there was any disagreement between the two reviewers on the included literature, it was resolved through discussion with the third reviewer.

### Data extraction

A table covering the study data was designed with reference to the Cochrane Handbook of Systematic Reviews (Higgins et al., 2022). Two researchers independently collected data from each study. The data collected included the name of the study lead author, year of publication, country, age, number of subjects, type and duration of intervention, assessment tool, and the data (mean ± SD) required for the meta‐analysis. If studies reported multiple self‐efficacy measures (e.g., both medication adherence efficacy and diabetes management efficacy), the most representative measure was selected from each study based on its primary focus and the content of the intervention to ensure data independence. Subgroup analyses were subsequently conducted according to the type of self‐efficacy.

### Assessment

The risk of bias was assessed using the Risk of Bias 2.0 tool from the Cochrane Handbook of Systematic Reviews, covering the following aspects: selection bias, performance bias, detection bias, attrition bias, reporting bias, and other biases (Higgins et al., 2022). Review Manager 5.4 was used to evaluate the risk of bias. The quality of the evidence was assessed using the GRADE profiler, which includes five levels: study limitations (risk of bias), inconsistencies, indirectness, imprecision, and publication bias (Guyatt et al., 2011). The third reviewer was consulted to resolve any discrepancies.

### Data analysis

This meta‐analysis was performed using RevMan 5.4 and Stata 18.0 software. Results were reported as mean difference (*MD*) if the outcome was measured on the same scale and as standard mean difference (*SMD*) otherwise. The size of the combined intervention effect was expressed as Cohen's *d*. A significance level of *p* < 0.05 was used. Heterogeneity between studies was assessed by calculating *I* ^2^. When *I* ^2^ > 50%, significant heterogeneity was indicated, and a random‐effects model was used. If *I* ^2^ ≤ 50%, a fixed‐effects model was used. Funnel plot and Egger's tests (using Stata 18.0) were used to explore publication bias (Egger et al., 1997). To further investigate potential sources of heterogeneity, this study conducted subgroup analyses and meta‐regression on the effect sizes of self‐efficacy, examining how factors such as intervention type, disease type, participant age, publication period, country, and income level influenced the estimated effects.

## RESULTS

**FIGURE 1:**
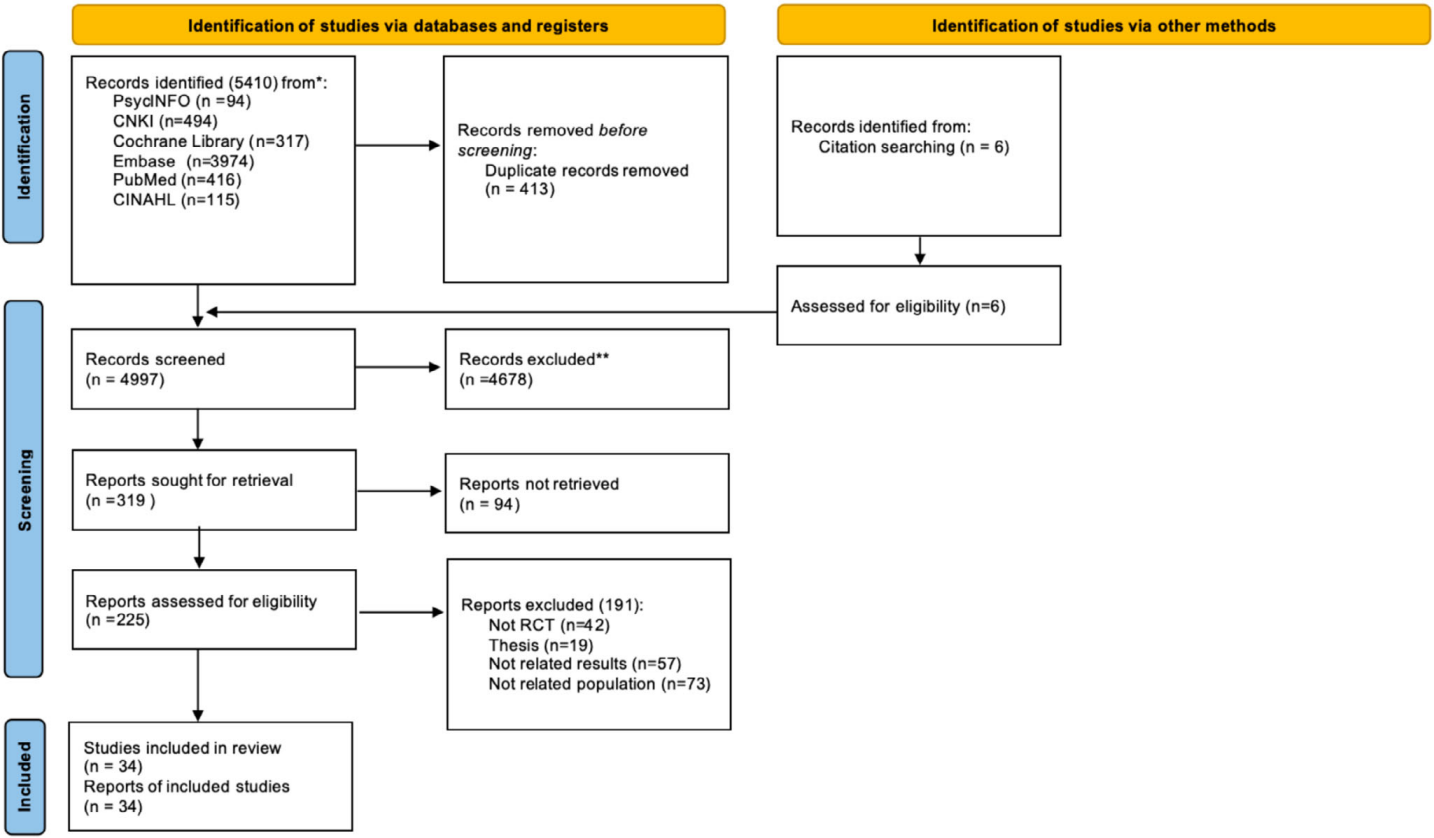
PRISMA flow chart.

After searching with the strategies, 5410 records were retrieved from the following databases: PsycINFO (94), CNKI (494), Cochrane Library (317), EMBASE (3974), PubMed (416), and CINAHL (115). Four hundred thirteen duplicate records were removed using EndNote software. An additional six articles were identified by reference search. Two reviewers screened the titles and abstracts according to the inclusion criteria, ultimately excluding 4678 articles. A total of 319 records were retained for full‐text search, of which 94 were unavailable. Finally, the remaining 225 articles were evaluated in detail, and 187 were excluded for reasons including non‐randomized controlled trials (42), theses (19), outcome irrelevance (57), and population irrelevance (73). Ultimately, 34 studies were included in the review and meta‐analysis (Andreae et al., 2021; Appalasamy, Joseph, et al., 2020; Appalasamy, Quek, et al., 2020; Barroso et al., 2014; Bell et al., 2021, p. 202; Campbell et al., 2015; Crogan et al., 2008; Cui et al., 2025; Dennick et al., 2015; Falzon et al., 2015; Feng, Malloch, et al., 2021; Feng, Shen, & Jin, 2021; Gao & Liu, 2022; Giesler et al., 2017; He et al., 2020; Huang, Xuan, et al., 2024; Iannello et al., 2018; Lee et al., 2006; Lely et al., 2022; Liu et al., 2020; Liu et al., 2022; Lopez‐Olivo, Des Bordes, et al., 2021; Lopez‐Olivo, Lin, et al., 2021; McCaughan et al., 2013; Song & Cheng, 2023; Tian et al., 2024; Wang et al., 2025; Yang, 2022; Zarifsaniey et al., 2022; Zhang et al., 2022; Zheng et al., 2024; Zhou et al., 2020; Zhu, Jia, et al., 2024; Zhu, Chen, et al., 2024). The PRISMA flowchart (Figure 1) illustrates the search process.

### Characteristics of included studies

The relevant features of the included studies were shown in Table S2. This systematic and meta‐analysis included 34 RCTs involving patients with a variety of chronic conditions, most commonly cancer (nine trials), diabetes (nine trials), and stroke (three trials), alongside other conditions such as HIV, arthritis, chronic pain, and post‐traumatic stress disorder (PTSD). Sample sizes ranged from 10 to 670 participants, with a total of 4584 individuals. The age range was broad, spanning from adolescence (mean 12.54 ± 3.46 years) to older adulthood (mean 79.67 ± 9.03 years).

The studies were geographically diverse, with China contributing the largest number (*n* = 16), followed by the United States (*n* = 8) and Malaysia (*n* = 2). Additional studies were conducted in Australia, Canada, France, Germany, Iran, Italy, the Netherlands, and the United Kingdom (*n* = 1 each). Publication years ranged from 2006 to 2025, with 25 studies published after 2020, reflecting sustained and growing scholarly interest in this field.

Narrative‐based interventions encompass a wide range of approaches. On the one hand, technology‐enabled methods include digital storytelling, narrative websites, and message‐based narratives. On the other hand, structured therapeutic practices led by health‐care professionals employ techniques such as problem externalization, deconstruction, identity reconstruction, and the collaborative creation of alternative stories. Narrative Exposure Therapy has been primarily applied in trauma populations, whereas narrative care delivered by health‐care professionals integrates emotional support with individualized narrative reconstruction, representing a particularly prominent model in clinical practice.

Across the included studies, both self‐efficacy and self‐management were assessed using self‐report measures. In terms of self‐efficacy, the assessment tools can be categorized into three major types. The most widely used are disease‐specific self‐efficacy scales, such as the Diabetes Management Self‐Efficacy Scale (DMSES), the Strategies Used by People to Promote Health (SUPPH), the Arthritis Self‐Efficacy Scale (ASES), the Exercise Self‐Efficacy Scale, the Epilepsy Self‐Management Scale (ESMS), and the Chronic Disease Self‐Efficacy Scale (CSES). In addition, the General Self‐Efficacy Scale (GSES) is applied to assess overall confidence, while medication‐specific self‐efficacy scales, including the Self‐Efficacy for Appropriate Medication Use Scale (SEAMS) and the Medication Understanding and Use Self‐Efficacy Scale (MUSE), are used to evaluate medication‐related confidence and adherence.

For self‐management, commonly adopted tools include the Summary of Diabetes Self‐Care Activities Scale (SDSCA), the Exercise of Self‐Care Agency Scale (ESCA), and disease‐specific instruments such as the Liver Cirrhosis Self‐Management Scale. Moreover, some studies employed comprehensive self‐management scales encompassing multiple domains to evaluate patients' actual behaviors in daily life, symptom control, treatment adherence, and social adaptation.

Intervention durations ranged from a single session to 6 months. Most studies evaluated outcomes over the short term (immediately post‐intervention) or medium term (1–6 months), while only a limited number included long‐term follow‐up beyond 6 months. Seven studies specifically assessed the effects during the follow‐up period after the intervention.

### Risk of bias in the included studies

**FIGURE 2:**
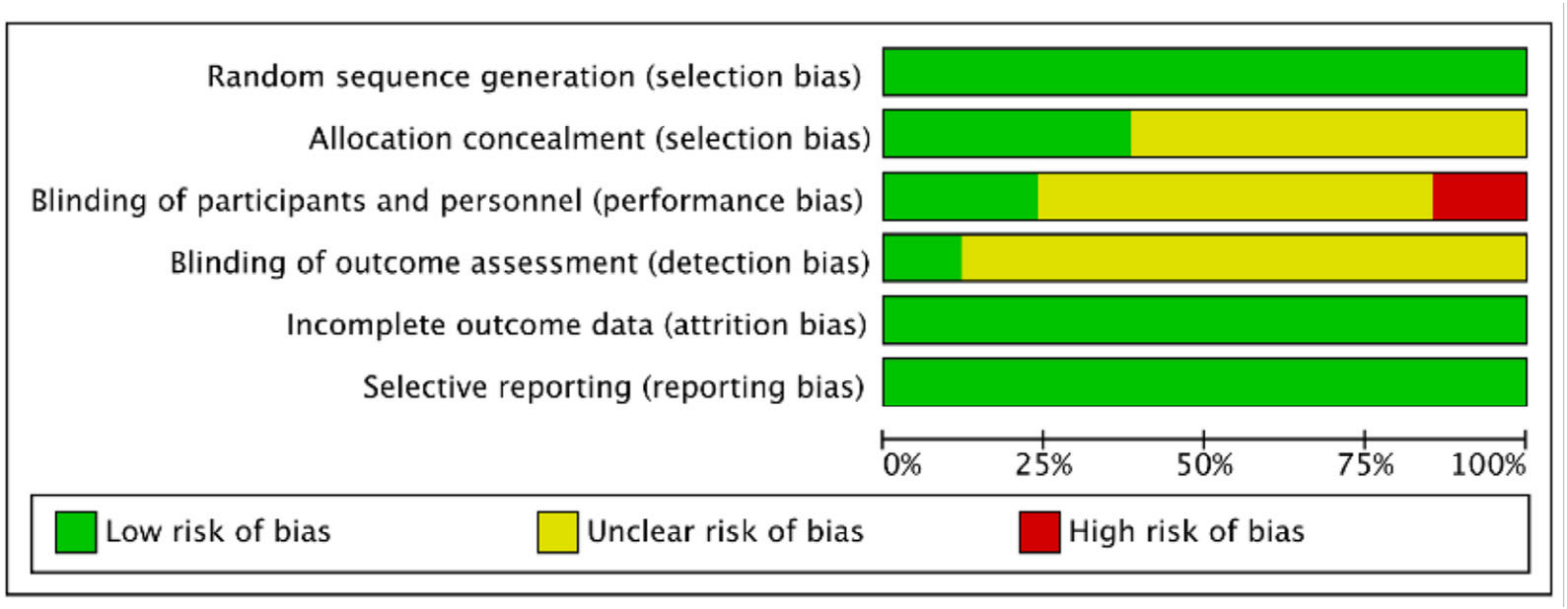
Risk of bias summary for included studies.

A summary of the risk of bias in the included studies was shown in Figures 2 and S1. All 34 studies demonstrated a low risk of bias in random sequence generation (selection bias) and outcome reporting (reporting bias), suggesting that the quality control in these methodological domains was relatively robust. In contrast, there was a considerable risk of bias regarding allocation concealment and blinding of outcome assessment: five studies explicitly reported not using blinding, 30 studies did not specify whether outcome assessors were blinded, and 21 studies did not report on allocation concealment (selection bias). The absence or insufficiency of methodological reporting in these areas constituted the primary source of bias in this review.

### Self‐efficacy

**FIGURE 3:**
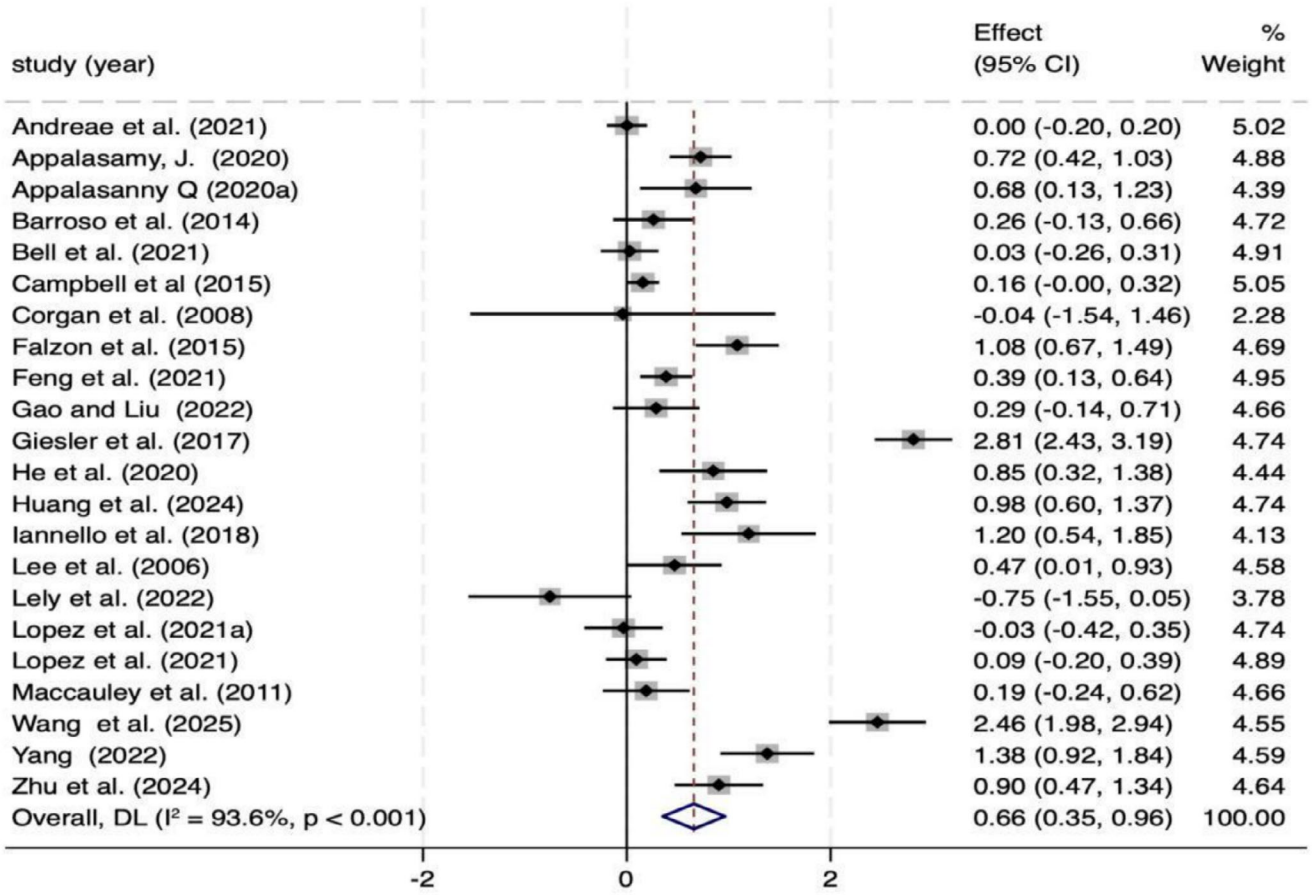
Forest plot of self‐efficacy effects across 22 studies.

This meta‐analysis included 22 studies comprising a total of 3152 patients with chronic diseases (1526 in the intervention group and 1626 in the control group). As shown in Figure 3, narrative‐based interventions were found to significantly improve self‐efficacy (*p* < .001). However, according to the GRADE framework, the certainty of the evidence for this outcome was rated as low. Despite observed heterogeneity, most studies consistently reported improvements in self‐efficacy, and the overall effect remained positive. Nevertheless, because of small sample sizes and methodological limitations in some studies, the overall reliability of the evidence was limited. Moreover, subgroup analysis indicated that the intervention effect diminished over time, showing significant short‐term benefits but no significant long‐term effects (*p* > .05; see Figure S2).

### Self‐management

**FIGURE 4:**
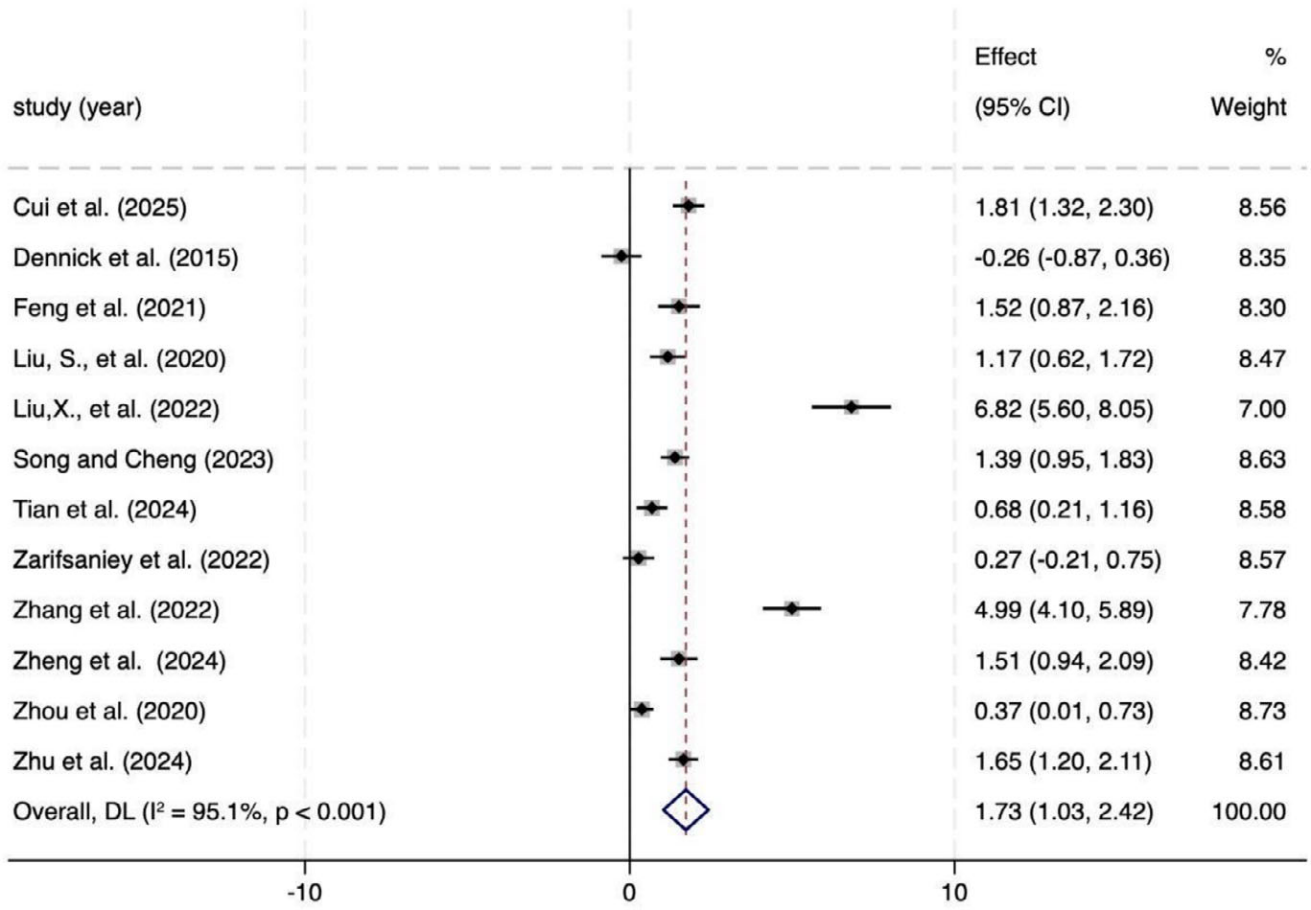
Forest plot of self‐management effects across 12 studies.

A meta‐analysis examining the effect of interventions on self‐management included 12 studies, with 458 participants in the intervention group and 453 in the control group. As shown in Figure 4, narrative‐based interventions significantly improved self‐management abilities in patients with chronic diseases (*p* < .001). However, substantial heterogeneity was observed across the studies. According to GRADE criteria, owing to this considerable heterogeneity and the potential risk of bias, the certainty of the evidence was rated as low.

### Subgroup analysis of self‐efficacy effects

The subgroup analysis revealed that the impact of narrative‐based interventions on self‐efficacy was significantly moderated by national economic level, patient age, and disease type, whereas no significant differences were observed across groups in terms of intervention format or duration (Table 1). Moreover, the meta‐regression analysis shows that the effect of national income level on the effect size approaches statistical significance (*p* = .055), whereas other variables—such as type of self‐efficacy, age, and intervention format—are not significant independent predictors. Detailed meta‐regression results are provided in Table S3.

**TABLE 1:** Subgroup analysis of narrative‐based interventions on self‐efficacy.

Variable	Subgroup	No. of studies	Sample size	Overall effects (*SMD*, 95% CI) (*p*‐value)	*I* ^*2*^ (%)	p‐value (*Q* test)	Subgroup differences(p‐value)
Country income‐level	High income	13	2472	0.39 (0.01–0.77) *p* = .044	94.3	*p* < .001	*p* = .019
	Upper to middle income	9	363	1.04 (0.65–0.31) *p* < .001	85.4	*p* < .001	
Age	< 44	3	403	1.40 (−0.34–3.15) *p* = .115	98.5	*p* < .001	*p* = .236
	45–59	12	2099	0.65 (0.33–0.97) *p* < .001	91.6	*p* < .001	
	> 60	7	333	0.31 (−0.06–0.68) *p* = .104	70.2	*p* = .003	
Intervention type	Interpersonal narrative	9	909	0.69 (0.08–1.30) *p* = .026	93.3	*p* < .001	*p* = .972
	Digital storytelling	11	1,496	0.65 (0.22–1.07) *p* = .003	94.7	*p* < .001	
	Narrative message	2	297	0.55 (−0.49–1.58) *p* = .301	94.2	*p* < .001	
Duration	Single or brief sessions(s)	5	629	0.35 (−0.05–0.75) *p* = .090	83.2	*p* < .001	*p* = .345
	Short‐term (1 day to 1 month)	11	1,673	0.80 (0.33–1.27) *p* = .001	94.6	*p* < .001	
	Long‐term (> 1 month to 6 months)	6	850	0.62 (−0.15–1.40) *p* = .113	95.3	*p* < .001	
Publication year (period)	2006–2019	8	1,221	0.80 (0.10–1.50) *p* = .025	96	*p* < .001	*p* = .569
	2020–2025	14	1931	0.58 (0.25–0.90) *p* = .001	91.4	*p* < .001	
Type of disease	Diabetes	4	1,311	0.62 (−0.05–1.29) *p* = .070	96.7	*p* < .001	*p* < .001
	Stroke	2	312	0.71 (0.45–0.98) *p* < .001	0.0	*p* = .884	
	Cancer	6	575	0.98 (0.10–1.85) *p* = .028	95.0	*p* < .001	
	Arthritis and pain	3	517	0.18 (−0.07–0.43) *p* = .164	48.9	*p* = .141	
	Others	5	231	0.38 (−0.13–0.88) *p* = .142	77.4	*p* = .001	
	Heart disease	2	206	1.16 (0.77–1.55) *p* < .001	40.3	*p* < .001	
Type of self‐efficacy	Medication‐specific self‐efficacy	4	874	0.45 (0.16–0.73) *p* = .002	73.1	*p* = .011	*p* = .499
	General self‐efficacy	9	634	0.82 (0.27–1.37) *p* = .004	89.7	*p* < .001	
	Disease‐specific self‐efficacy	10	2048	0.54 (0.09–1.00) *p* = .019	95.7	*p* < .001	

### Subgroup analysis of self‐management effects

The subgroup analysis of the self‐management effect based on narrative‐based intervention revealed significant heterogeneity in intervention outcomes, requiring cautious interpretation (see Table 2). The findings indicated that intervention duration, intervention type, disease type, and publication year were associated with differences in intervention effects. However, no significant differences were observed across age groups. Although the subgroup analysis suggested that certain variables might be related to the intervention effect, the multivariate meta‐regression analysis—after further examining the independent predictive roles of these variables—found that age, disease type, intervention type, intervention duration, and publication year did not have significant predictive effects on the self‐management outcomes of narrative intervention (*p* > .05) (Table S4).

**TABLE 2:** Subgroup analysis of narrative‐based interventions on self‐management.

Variable	Subgroup	No. of studies	Sample size	Overall effects (*SMD*, 95% CI) (*p*‐value)	*I* ^*2*^(%)	*p*‐value (*Q* test)	Subgroup differences (*p*‐value)
Age	< 45	5	390	2.16 (0.93–3.39) *p* < .001	96	*p* = .001	*p* = .439
	45–59	4	328	1.24 (0.54–1.95) *p* < .001	87.9	*p* = .001	
	> 60	3	193	1.78 (−0.75–4.31) *p* < .001	97.9	*p* = .167	
Intervention type	Interpersonal narrative	10	804	2.08 (1.32–2.84) *p* < .001	95.1	*p* < .001	*p* < .001
	Digital storytelling	1	66	−0.27 (−0.21–0.75) *p* = .275	0.0		
	Writing narrative	1	41	−0.26 (−0.87–0.36) *p =* .419	0.0		
Duration	Short‐term (≤ 1 month)	4	280	2.33 (1.02–3.64) *p* < .001	94.4	*p* < .001	*p* = .003
	Medium‐term (2–3 months)	11	359	0.52 (0.03–1.00) *p* = .037	79.5	*p* = .001	
	Long‐term (≥ 6 months)	6	272	3.16 (1.18–5.15) *p* = .002	97.1	*p* < .001	
Publication year (period)	2024–2025	4	324	1.41 (0.90–1.92) *p* < .001	76.5	*p* = .005	*p* = .012
	2015–2020	3	221	0.44 (−0.27–1.14) *p* = .224	83	*p* = .003	
	2021–2023	5	366	2.93 (1.15–4.71) *p =* .001	97.4	*p* < .001	
Type of disease	Cancer	3	232	2.73 (1.01–4.45) *p* = .002	95.6	*p* < .001	*p* = .003
	Diabetes	5	287	0.67 (0.11–1.23) *p* = .019	80.9	*p* < .001	
	Neurological disorders	3	272	3.16 (1.18–5.15) *p* = .002	97.1	*p* < .001	
	Other chronic conditions	1	120	0.37 (0.01–0.73) *p* = .044	0.0		

### Publication bias and sensitivity analysis

#### Self‐efficacy

**FIGURE 5:**
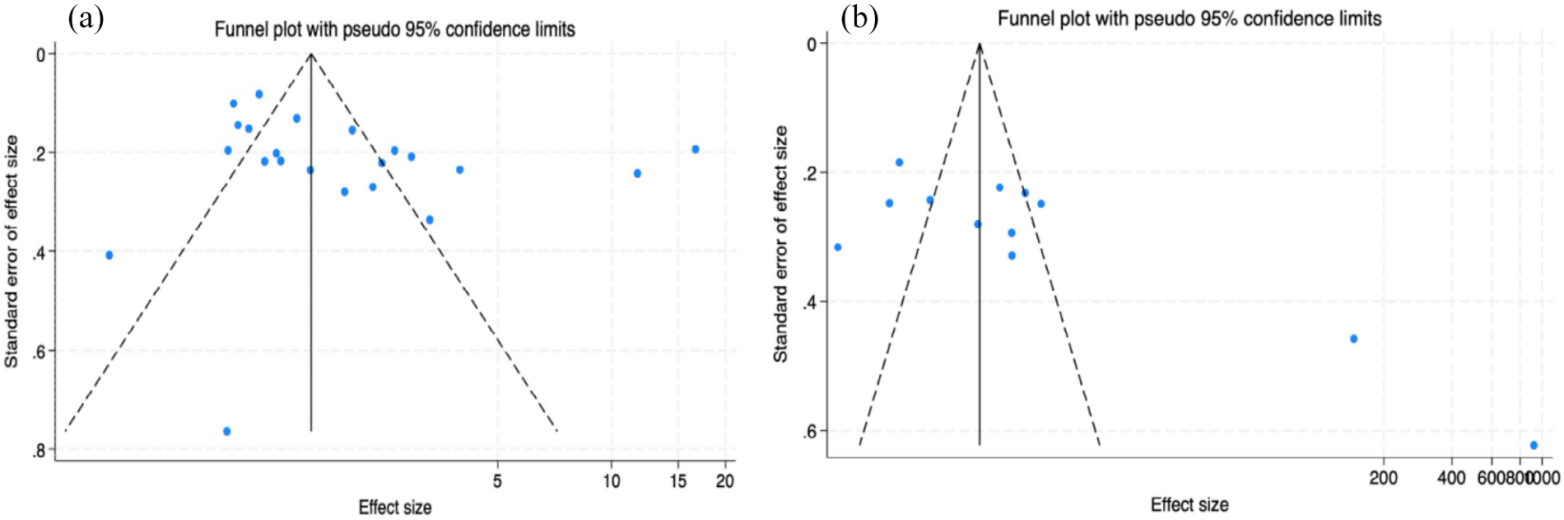
Funnel plot for studies on self‐efficacy and self‐management.

The funnel plot (Figure 5a) indicates that there may be some degree of publication bias because the distribution of points on both sides of the plot was not completely symmetric, and there were some outliers. According to Egger's test, no significant publication bias was found (*t* = 1.83, *p* = .082), which indicates that there was no obvious systematic bias. The leave‐one‐out sensitivity analysis revealed that excluding any single study resulted in combined effect sizes (*SMD*) ranging consistently from 0.55 to 0.71, closely aligning with the overall effect size (*SMD* = 0.66). Moreover, all corresponding confidence intervals excluded 0, indicating that the overall pooled effect size estimate was robust. Details were shown in Tables S5 and S7.

#### Self‐management

The funnel plot appeared asymmetrical (see Figure 5b), and Egger's test indicated potential publication bias (*p* = .007); however, meta‐analysis without imputing missing studies did not alter the combined effect size. Sensitivity analysis revealed that the overall conclusion was slightly influenced by individual studies, particularly Liu et al. (2020) and Zhang et al. (2022), with the combined effect size ranging from 1.33 to 1.91. Overall, despite minor indications of publication bias and slight dependence on specific studies, the evidence supports a positive effect of the intervention. Details were shown in Supplementary Table S6 and Table S8.

## DISCUSSION

This systematic review and meta‐analysis examined the effects of narrative‐based interventions on self‐efficacy and self‐management in patients with chronic diseases. The findings showed that narrative‐based interventions significantly improved both outcomes; however, substantial heterogeneity was present across studies, and some results were sensitive to individual trials. Consequently, the certainty of the evidence is limited, and the conclusions should be interpreted with caution.

### Effects of self‐efficacy

Narrative‐based interventions can significantly enhance the self‐efficacy of patients with chronic diseases. This supports the core mechanism whereby narratives promote cognitive reconstruction through role identification and emotional resonance (Georgiadis & Johnson, 2023; Gurney et al., 2023). However, the sustainability of these effects remains a key concern, which aligns with earlier findings indicating that narrative interventions primarily yield short‐ to mid‐term benefits (Georgiadis & Johnson, 2023). The emotional activation and immediate cognitive shifts elicited by narratives may naturally diminish without ongoing reinforcement. In addition, the disease experiences and psychological needs of patients with chronic illnesses are dynamic, and static narrative content may fail to align with their evolving life contexts, thereby compromising the stability of long‐term effects (Gucciardi et al., 2016). Despite this time‐dependent attenuation, the subgroup analysis (Table 1) found no significant differences across types of interventions, suggesting that narrative efficacy is driven more by shared cognitive–emotional mechanisms than by specific modes of delivery.

This systematic review and meta‐analysis also observed differences in the point estimates of effects across national income levels and disease types. National income level may reflect variations in health system structures, cultural contexts, medical accessibility, and patient needs, all of which could influence the adaptability and acceptability of narrative content in different settings (Abel et al., 2024; Metanmo et al., 2024; Naderbagi et al., 2024). For example, this numerical trend may be partially explained by the combination of limited systemic resources in upper‐ to middle‐income countries and high patient demand, where traditional health‐care systems often provide insufficient psychological and social support (Acharibasam & Wynn, 2018; Galagali & Brooks, 2020; Pham et al., 2020). In such settings narrative‐based interventions—being low‐cost and easy to implement—may be more appealing (Loy & Kowalsky, 2024; Ranjit et al., 2015), whereas in high‐income countries with diverse cultures and complex health‐care systems, insufficient cultural adaptation of narrative materials may attenuate their effectiveness (Larkey & Hecht, 2010; Murphy et al., 2013). Similarly, higher point estimates were observed among patients with severe chronic conditions, possibly because of their greater psychological needs, more complex self‐management demands, and heightened perceptions of health threats, which may enhance emotional resonance with narrative content (Lévai et al., 2024).

### Effects of self‐management

Compared with self‐efficacy, the overall effect of narrative‐based interventions on self‐management behaviors was larger, yet the heterogeneity among studies was extremely high. This suggests that behavioral change is inherently more complex than psychological perception, with outcomes shaped by both contextual factors and individual‐level differences. Although the pooled effect was statistically significant, the substantial heterogeneity indicates that the findings should be interpreted with caution.

The subgroup analysis indicated that multiple study characteristics significantly moderated the effects of narrative‐based interventions on self‐management. First, intervention type emerged as the most critical influencing factor. Interpersonal narrative interventions showed the strongest effect, suggesting a stable and reliable impact. This advantage may stem from the immediate social feedback and stronger emotional resonance inherent in interactive contexts, which can enhance participants' engagement and meaning‐making processes (Kleinbub et al., 2020). In contrast, digital storytelling and writing narratives did not demonstrate significant effects, indicating that narrative formats lacking interactive components may have limited influence on self‐management, and their underlying mechanisms warrant further investigation (Perski et al., 2017).

Second, the duration of intervention differed significantly across subgroups, showing a clear “U‐shaped” pattern: both short‐term interventions (≤1 month) and long‐term interventions (≥6 months) yielded large effect sizes, whereas medium‐term interventions (2–3 months) showed markedly attenuated effects. This suggests that narrative interventions may exert strong early effects through emotional activation and motivational priming, while long‐term benefits may stem from sustained exposure and repeated meaning reconstruction. In contrast, a temporary “effect plateau” may emerge during the medium‐term period (Oschatz & Marker, 2020).

Moreover, this effect is observed across various disease types; its magnitude may differ, potentially reflecting variations in how different conditions influence patients' self‐identity and the depth of emotional resonance elicited by the narrative content (Lévai et al., 2024). Publication year also acted as a moderating factor. Subgroup analysis revealed temporal variations, with more recent studies (2021–2025) generally reporting stronger effect sizes compared with earlier studies (2015–2020). This trend may reflect improvements in intervention design and implementation quality over time. Additionally, age did not serve as a significant moderator, suggesting that the core mechanisms of narrative interventions—grounded in stories that evoke emotional and cognitive processing—may be universally effective across age groups.

### Analysis of the potential sources of heterogeneity

The meta‐regression analysis indicated that all the preset variables did not emerge as significant independent predictors after controlling for other factors, contrasting with some significant inter‐group differences observed in the subgroup analysis. This inconsistency may reflect the synergistic, multi‐factorial nature of narrative intervention effects, where the independent contribution of a single variable is diluted by complex interactions. It also highlights the potential role of unmeasured key moderators, such as the quality of narrative content, cultural adaptability, patient engagement, and immersion. Furthermore, given the limited number of included studies, the statistical power of the meta‐regression may be insufficient, representing an area for improvement in future research.

Although the funnel plot and Egger test suggested the possibility of publication bias (especially in self‐management outcomes), the leave‐one‐out sensitivity analysis indicated that the combined results were highly robust. The consistency of self‐efficacy improvement was relatively high, while self‐management outcomes were more sensitive to individual studies, such as Liu et al. (2020) and Zhang et al. (2022), indicating that behavioral transformation may be more influenced by situational and individual factors compared to psychological perception.

### Strengths and limitations

The strength of this study lies in its systematic inclusion of multiple RCTs and its simultaneous evaluation of two key outcomes—self‐efficacy and self‐management behavior. This approach provides a comprehensive overview of the evidence regarding narrative interventions in chronic disease management. The study adhered rigorously to methodological standards for systematic review and meta‐analysis, incorporating multiple layers of sensitivity and subgroup analyses to enhance the transparency and robustness of the findings.

However, this systematic review and meta‐analysis also has several limitations. The methodological quality of the included trials varies considerably, with many failing to report essential details such as randomization procedures, allocation concealment, and blinding. In addition, substantial heterogeneity exists across studies, likely stemming from variations in intervention content, implementation fidelity, and outcome measurement tools. Finally, most studies were conducted within similar cultural contexts, which limits the cross‐cultural generalizability of the findings.

### Future research directions

Future research should focus on enhancing the scientific rigor and clinical translational value of narrative‐based interventions and directly address the main limitations identified in this review, including variable methodological quality, insufficient intervention standardization, lack of mechanism‐focused variables, and short follow‐up periods. A primary task is to improve methodological quality and reporting transparency. Future studies should rigorously adhere to guidelines such as CONSORT, comprehensively report key aspects including randomization, blinding, and implementation processes, and provide standardized descriptions of intervention content and procedures. Core elements—such as narrative material development, cultural adaptation, storytelling methods, and interaction structures—should be systematically reported, and scalable intervention frameworks should be explored.

In addition, research should move beyond demographic variables to investigate process‐related factors, including narrative quality, emotional immersion, and patient engagement. Both theoretical and empirical studies indicate that highly immersive narratives enhance cognitive and emotional involvement, thereby facilitating behavioral change (Oschatz & Marker, 2020; Shaffer et al., 2018). Mixed‐methods and multi‐level designs can help elucidate the mechanisms of intervention and identify the populations for whom they are most effective. Furthermore, cross‐cultural and cross‐disease multi‐center studies are needed, with particular attention to applicability in disadvantaged groups and resource‐limited settings. Intervention designs should incorporate longer follow‐up periods and dynamic assessment strategies to capture the nonlinear trajectory of narrative effects. Digital innovations can also be integrated to enhance interactivity, immersion, and personalized adaptation, exploring novel intervention formats, such as virtual reality and artificial intelligence‐based narrative assistants, thereby optimizing both effectiveness and accessibility.

## CONCLUSION

This systematic review and meta‐analysis indicate that narrative‐based interventions can improve the self‐efficacy and self‐management behaviors of patients with chronic diseases. The evidence shows that their effect on self‐efficacy is significant but may be more limited to the short term, whereas their effect on self‐management is larger yet highly variable and influenced by intervention type and duration. However, these conclusions are constrained by several important factors. The overall quality of evidence is low, with major limitations including substantial heterogeneity across studies, methodological weaknesses in the original trials, and a potential risk of publication bias—particularly for self‐management outcomes. Therefore, the current findings should be interpreted with caution. Narrative‐based interventions, as an adjunct strategy for chronic disease management, demonstrate promising clinical potential, especially when delivered in an interactive format and supported by sustained engagement. To translate this potential into robust clinical practice, future research must prioritize methodological rigor, investigate underlying mechanisms of action, and develop dynamic, personalized, and culturally responsive intervention models.

## CONFLICT OF INTEREST STATEMENT

No potential conflict of interest was reported by the authors.

## FUNDING INFORMATION

The authors reported that there is no funding associated with the work featured in this article.

Systematic Review Register (PROSPERO).

CRD42023413162.

## ETHICS STATEMENT

This study is a systematic review and meta‐analysis of previously published literature and does not involve direct contact with human or animal subjects. Therefore, ethical approval was not required.

## Supporting information


**Table S1.** Search strategies.
**Table S2.** Characteristics of Included Studies.
**Table S3.** Summary of meta‐regression results.
**Table S4.** Results of leave‐one‐out method in sensitivity analysis.
**Table S5.** Results of leave‐one‐out method in sensitivity analysis.
**Figure S1.** Risk of Bias Assessment of 34 Included Studies.
**Figure S2.** Egger's Test for Publication Bias in a Meta‐Analysis for Self‐Efficacy.
**Figure S3.** Egger's Test for Publication Bias in a Meta‐Analysis for Self‐Management.

## Data Availability

This study is a systematic review and meta‐analysis. All data are derived from published research literature. The data extracted and analyzed in this study are included in the article and its Supporting Information. All original studies used to support the findings of this study can be obtained by searching the databases mentioned in the text.

## References

[aphw70127-bib-0001] Abel, Z. D. V. , Roope, L. S. J. , Duch, R. , & Clarke, P. M. (2024). Access to healthcare services during the COVID‐19 pandemic: A cross‐sectional analysis of income and user‐access across 16 economically diverse countries. BMC Public Health, 24(1), 2678. 10.1186/s12889-024-20147-y 39350210 PMC11443786

[aphw70127-bib-0002] Acharibasam, J. W. , & Wynn, R. (2018). Telemental health in low‐ and middle‐income countries: A systematic review. International Journal of Telemedicine and Applications, 2018, 1–10). Hindawi Publishing Corporation. 10.1155/2018/9602821 PMC6241375 30519259

[aphw70127-bib-0003] Anderson, E. , & Durstine, J. L. (2019). Physical activity, exercise, and chronic diseases: A brief review. Sports Medicine and Health Science, 1(1), 3–10. 10.1016/j.smhs.2019.08.006 35782456 PMC9219321

[aphw70127-bib-0004] Andreae, S. J. , Andreae, L. J. , Cherrington, A. L. , Richman, J. S. , Johnson, E. , Clark, D. , & Safford, M. M. (2021). Peer coach delivered storytelling program improved diabetes medication adherence: A cluster randomized trial. Contemporary Clinical Trials, 104, 106358. 10.1016/j.cct.2021.106358 33737200

[aphw70127-bib-0005] Appalasamy, J. R. , Joseph, J. P. , Seeta Ramaiah, S. , Md Zain, A. Z. , Quek, K. F. , & Tha, K. K. (2020). Video narratives intervention among stroke survivors: Feasibility and acceptability study of a randomized controlled trial. JMIR Aging, 3(2), e17182. 10.2196/17182 32469839 PMC7382013

[aphw70127-bib-0006] Appalasamy, J. R. , Quek, K. F. , Md Zain, A. Z. , Joseph, J. P. , Seeta Ramaiah, S. , & Tha, K. K. (2020). An evaluation of the video narrative technique on the self‐efficacy of medication understanding and use among post‐stroke patients: A randomized‐controlled trial. Patient Preference and Adherence, 14, 1979–1990. 10.2147/PPA.S253918 33116441 PMC7585263

[aphw70127-bib-0007] Bandura, A. (1977). Self‐efficacy: Toward a unifying theory of behavioral change. Psychological Review, 84(2), 191–215. 847061 10.1037//0033-295x.84.2.191

[aphw70127-bib-0008] Bandura, A. (1989). Human agency in social cognitive theory. American Psychologist, 44(9), 1175–1184. 10.1037/0003-066x.44.9.1175 2782727

[aphw70127-bib-0009] Barroso, J. , Relf, M. V. , Williams, M. S. , Arscott, J. , Moore, E. D. , Caiola, C. , & Silva, S. G. (2014). A randomized controlled trial of the efficacy of a stigma reduction intervention for HIV‐infected women in the deep south. AIDS Patient Care and STDs, 28(9), 489–498. 10.1089/apc.2014.0014 25084499 PMC4135326

[aphw70127-bib-0010] Bell, T. , Noar, S. M. , & Lazard, A. J. (2021). Narrative vs. standard of care messages: Testing how communication can positively influence adolescents with type 1 diabetes. Journal of Health Communication, 26(9), 626–635. 10.1080/10810730.2021.1985657 34649469

[aphw70127-bib-0011] Benzo, R. P. (2024). Self‐management programs and the pursuit of behavior change. Respiratory Care, 69(6), 678–685. 10.4187/respcare.11987 38806226 PMC11147631

[aphw70127-bib-0012] Boger, E. , Ellis, J. , Latter, S. , Foster, C. , Kennedy, A. , Jones, F. , Fenerty, V. , Kellar, I. , & Demain, S. (2015). Self‐management and self‐management support outcomes: A systematic review and mixed research synthesis of stakeholder views. PLoS One, 10(7) Public Library of Science, e0130990. 10.1371/journal.pone.0130990 26162086 PMC4498685

[aphw70127-bib-0013] Campbell, T. , Dunt, D. , Fitzgerald, J. L. , & Gordon, I. (2015). The impact of patient narratives on self‐efficacy and self‐care in Australians with type 2 diabetes: Stage 1 results of a randomized trial. Health Promotion International, 30(3), 438–448. 10.1093/heapro/dat058 23985247

[aphw70127-bib-0014] Cates, J. R. , Francis, D. B. , Ramirez, C. , Brown, J. D. , Schoenbach, V. J. , Fortune, T. , Powell Hammond, W. , & Adimora, A. A. (2015). Reducing concurrent sexual partnerships among blacks in the rural southeastern United States: Development of narrative messages for a radio campaign. Journal of Health Communication, 20(11), 1264–1274. 10.1080/10810730.2015.1018643 26134387 PMC4639399

[aphw70127-bib-0015] Chan, S. W.‐C. (2021). Chronic disease management, self‐efficacy and quality of life. Journal of Nursing Research, 29(1), e129. 10.1097/JNR.0000000000000422 PMC7808345 33427791

[aphw70127-bib-0016] Chen, J. , Tian, Y. , Yin, M. , Lin, W. , Tuersun, Y. , Li, L. , Yang, J. , Wu, F. , Kan, Y. , & Li, X. (2023). Relationship between self‐efficacy and adherence to self‐management and medication among patients with chronic diseases in China: A multicentre cross‐sectional study. Journal of Psychosomatic Research, 164, 111105. 10.1016/j.jpsychores.2022.111105 36495756

[aphw70127-bib-0017] Christiansen, J. , Lund, R. , Qualter, P. , Andersen, C. M. , Pedersen, S. S. , & Lasgaard, M. (2021). Loneliness, social isolation, and chronic disease outcomes. Annals of Behavioral Medicine, 55(3), 203–215. 10.1093/abm/kaaa044 32865550

[aphw70127-bib-0018] Crogan, N. L. , Evans, B. C. , & Bendel, R. (2008). Storytelling intervention for patients with cancer: Part 2—Pilot testing. Oncology Nursing Forum, 35(2), 265–272. 10.1188/08.ONF.265-272 18321839

[aphw70127-bib-0019] Cui, W. , Zhu, H. , & Yan, K. (2025). Effects of cox‐based staged narrative therapy on self‐management in gastric cancer patients. China Medical Innovation, 22, 85–89.

[aphw70127-bib-0020] Dennick, K. , Bridle, C. , & Sturt, J. (2015). Written emotional disclosure for adults with type 2 diabetes: A primary care feasibility study. Primary Health Care Research & Development, 16, 179–187. 10.1017/S1463423614000188 24801108

[aphw70127-bib-0021] Devan, H. , Hale, L. , Hempel, D. , Saipe, B. , & Perry, M. A. (2018). What works and does not work in a self‐management intervention for people with chronic pain? Qualitative systematic review and meta‐synthesis. Physical Therapy, 98(5), 381–397. 10.1093/ptj/pzy029 29669089

[aphw70127-bib-0022] Duda‐Sikuła, M. , & Kurpas, D. (2024). Enhancing chronic disease management: Personalized medicine insights from rural and urban general practitioner practices. Journal of Personalized Medicine, 14(7), 706. 10.3390/jpm14070706 39063960 PMC11277769

[aphw70127-bib-0023] Egger, M. , Smith, G. D. , Schneider, M. , & Minder, C. (1997). Bias in meta‐analysis detected by a simple, graphical test. BMJ (Clinical Research Ed.), 315(7109), 629–634. 10.1136/bmj.315.7109.629 PMC2127453 9310563

[aphw70127-bib-0024] Falzon, C. , Radel, R. , Cantor, A. , & d'Arripe‐Longueville, F. (2015). Understanding narrative effects in physical activity promotion: The influence of breast cancer survivor testimony on exercise beliefs, self‐efficacy, and intention in breast cancer patients. Supportive Care in Cancer, 23(3), 761–768. 10.1007/s00520-014-2422-x 25186211

[aphw70127-bib-0025] Farley, H. (2020). Promoting self‐efficacy in patients with chronic disease beyond traditional education: A literature review. Nursing Open, 7(1), 30–41. 10.1002/nop2.382 31871689 PMC6917929

[aphw70127-bib-0026] Feng, B. , Malloch, Y. Z. , Kravitz, R. L. , Verba, S. , Iosif, A.‐M. , Slavik, G. , & Henry, S. G. (2021). Assessing the effectiveness of a narrative‐based patient education video for promoting opioid tapering. Patient Education and Counseling, 104(2), 329–336. 10.1016/j.pec.2020.08.019 32900605 PMC7855718

[aphw70127-bib-0027] Feng, X. H. , Shen, S. J. , & Jin, G. J. (2021). Effect of narrative nursing model on self‐management ability and quality of life of patients with advanced pancreatitis and diabetes. World Chinese Journal of Digestology, 29, 1316–1322. 10.11569/wcjd.v29.i22.1316

[aphw70127-bib-0028] Galagali, P. M. , & Brooks, M. J. (2020). Psychological care in low‐resource settings for adolescents. Clinical Child Psychology and Psychiatry, 25(3), 698–711. 10.1177/1359104520929741 32567351 PMC9137117

[aphw70127-bib-0029] Gao, C. , & Liu, Y. (2022). Application of narrative medicine‐guided psychological intervention in the self‐efficacy of patients undergoing radical lung cancer resection and their family caregivers. Guizhou Medicine, 46, 1992–1993.

[aphw70127-bib-0030] Gauci, J. , Bloomfield, J. , Lawn, S. , Towns, S. , & Steinbeck, K. (2021). Effectiveness of self‐management programmes for adolescents with a chronic illness: A systematic review. Journal of Advanced Nursing, 77(9), 3585–3599. 10.1111/jan.14801 33630315

[aphw70127-bib-0031] Georgiadis, E. , & Johnson, M. I. (2023). Incorporating personal narratives in positive psychology interventions to manage chronic pain. Frontiers in Pain Research, 4, 1253310. 10.3389/fpain.2023.1253310 37869366 PMC10588179

[aphw70127-bib-0032] Gesser‐Edelsburg, A. (2021). Using narrative evidence to convey health information on social media: The case of COVID‐19. Journal of Medical Internet Research, 23(3), e24948. 10.2196/24948 33674257 PMC7962859

[aphw70127-bib-0033] Giesler, J. M. , Keller, B. , Repke, T. , Leonhart, R. , Weis, J. , Muckelbauer, R. , Rieckmann, N. , Müller‐Nordhorn, J. , Lucius‐Hoene, G. , & Holmberg, C. (2017). Effect of a website that presents patients' experiences on self‐efficacy and patient competence of colorectal cancer patients: Web‐based randomized controlled trial. Journal of Medical Internet Research, 19(10), e334. 10.2196/jmir.7639 29030329 PMC5660297

[aphw70127-bib-0034] González‐Conde, V. M. , Pérez‐Fernández, V. , Ruiz‐Esteban, C. , & Valverde‐Molina, J. (2019). Impacto de la autoeficacia en la calidad de vida de niños con asma y sus cuidadores. Archivos De Bronconeumología, 55(4), 189–194. 10.1016/j.arbres.2018.07.008 30119934

[aphw70127-bib-0035] Gucciardi, E. , Jean‐Pierre, N. , Karam, G. , & Sidani, S. (2016). Designing and delivering facilitated storytelling interventions for chronic disease self‐management: A scoping review. BMC Health Services Research, 16(1), 249. 10.1186/s12913-016-1474-7 27401836 PMC4940988

[aphw70127-bib-0036] Gurney, L. , Chung, V. , MacPhee, M. , Chan, E. , Snyman, C. , Robinson, J. , Bertoli‐Haley, S. , & Baron, E. (2023). Exploring the impact of storytelling for hospitalized patients recovering from COVID‐19. Healthcare (Basel), 11(4), 589. 10.3390/healthcare11040589 36833123 PMC9957174

[aphw70127-bib-0037] Guyatt, G. , Oxman, A. D. , Vist, G. E. , Kunz, R. , Brożek, J. , Alonso‐Coello, P. , Montori, V. M. , Akl, E. A. , Djulbegovic, B. , Falck‐Ytter, Y. , Norris, S. L. , Williams, J. W. , Atkins, D. , Meerpohl, J. J. , & Schünemann, H. J. (2011). Grade guidelines: 4. Rating the quality of evidence—Study limitations (risk of bias). Journal of Clinical Epidemiology, 64(4), 407–415. 10.1016/j.jclinepi.2010.07.017 21247734

[aphw70127-bib-0038] He, S. , Li, J. , Luo, L. , & Wang, X. (2020). Effects of personalized narrative nursing on lung function and self‐efficacy in elderly patients with chronic obstructive pulmonary disease. Journal of Practical Medical Technology, 27, 1408–1410. 10.19522/j.cnki.1671-5098.2020.10.063

[aphw70127-bib-0039] Higgins, J. , Thomas, J. , Chandler, J. , Cumpston, M. , Li, T. , Page, M. , & Welch, V. (2022). Cochrane handbook for systematic reviews of interventions version 6.3. 2022. URL: https://Training.Cochrane.Org/Handbook/Current.

[aphw70127-bib-0040] Hinyard, L. , & Kreuter, M. W. (2006). Using narrative communication as a tool for health behavior change: A conceptual, theoretical, and empirical overview. Health Education & Behavior, 34(5), 777–792. 10.1177/1090198106291963 17200094

[aphw70127-bib-0041] Huang, J. , Xuan, L. , Zhang, N. , Zhang, Q. , Wang, Y. , Liu, Y. , & Zhou, T. (2024). Effects of improved narrative nursing based on the COM‐B model on quality of life and self‐efficacy in patients with coronary heart disease undergoing PCI. Journal of Bengbu Medical College, 49, 1651–1655. 10.13898/j.cnki.issn.1000-2200.2024.12.022

[aphw70127-bib-0042] Huang, Y. , Li, S. , Lu, X. , Chen, W. , & Zhang, Y. (2024). The effect of self‐management on patients with chronic diseases: A systematic review and meta‐analysis. Healthcare, 12, 2151. 10.3390/healthcare12212151 39517362 PMC11544912

[aphw70127-bib-0043] Iannello, P. , Biassoni, F. , Bertola, L. , Antonietti, A. , Caserta, V. A. , & Panella, L. (2018). The role of autobiographical story‐telling during rehabilitation among hip‐fracture geriatric patients. Europe's Journal of Psychology, 14(2), 424–443. 10.5964/ejop.v14i2.1559 PMC6016034 30008955

[aphw70127-bib-0044] Iovino, P. , Uchmanowicz, I. , & Vellone, E. (2024). Self‐care: An effective strategy to manage chronic diseases. Advances in Clinical and Experimental Medicine, 33(8), 767–771. 10.17219/acem/191102 39194160

[aphw70127-bib-0045] Jiang, X. , Wang, J. , Lu, Y. , Jiang, H. , & Li, M. (2019). Self‐efficacy‐focused education in persons with diabetes: A systematic review and meta‐analysis. Psychology Research and Behavior Management, 12, 67–79. 10.2147/PRBM.S192571 30774486 PMC6357887

[aphw70127-bib-0046] Kim, S. J. , Park, M. , & Song, R. (2021). Effects of self‐management programs on behavioral modification among individuals with chronic disease: A systematic review and meta‐analysis of randomized trials. PLoS One, 16(7) Public Library of Science, e0254995. 10.1371/journal.pone.0254995 34297741 PMC8301623

[aphw70127-bib-0047] Kleinbub, J. R. , Mannarini, S. , & Palmieri, A. (2020). Interpersonal biofeedback in psychodynamic psychotherapy. Frontiers in Psychology, 11, 1655. 10.3389/fpsyg.2020.01655 32849011 PMC7418492

[aphw70127-bib-0048] Laing, C. M. , Moules, N. J. , Estefan, A. , & Lang, M. (2017). Stories that heal: Understanding the effects of creating digital stories with pediatric and adolescent/young adult oncology patients. Journal of Pediatric Oncology Nursing, 34, 272–282. 10.1177/1043454216688639 28614999

[aphw70127-bib-0049] Larkey, L. K. , & Hecht, M. (2010). A model of effects of narrative as culture‐centric health promotion. Journal of Health Communication, 15(2), 114–135. 10.1080/10810730903528017 20390982

[aphw70127-bib-0050] Lee, H. , Fawcett, J. , & DeMarco, R. F. (2015). Storytelling/narrative theory to address health communication with minority populations. Applied Nursing Research, 30, 58–60. 10.1016/j.apnr.2015.09.004 27091254

[aphw70127-bib-0051] Lee, V. , Robin Cohen, S. , Edgar, L. , Laizner, A. M. , & Gagnon, A. J. (2006). Meaning‐making intervention during breast or colorectal cancer treatment improves self‐esteem, optimism, and self‐efficacy. Social Science & Medicine, 62(12), 3133–3145. 10.1016/j.socscimed.2005.11.041 16413644

[aphw70127-bib-0052] Lely, J. C. G. , Ter Heide, F. J. J. , Moerbeek, M. , Knipscheer, J. W. , & Kleber, R. J. (2022). Psychopathology and resilience in older adults with posttraumatic stress disorder: A randomized controlled trial comparing narrative exposure therapy and present‐centered therapy. European Journal of Psychotraumatology, 13(1), 2022277. 10.1080/20008198.2021.2022277 35126882 PMC8815622

[aphw70127-bib-0053] Lévai, T. , Lázár, G. , Krajinovic, E. , Devosa, I. , & Látos, M. (2024). Examining illness narratives in the context of the postoperative psychological state: A mixed‐methods study of emotion‐focused illness narrative. BioPsychoSocial Medicine, 18(1), 21. 10.1186/s13030-024-00318-4 39395999 PMC11470729

[aphw70127-bib-0054] Liu, S. , Peng, W. , & Wu, M. (2020). Effects of narrative nursing on self‐management and dietary behavior in patients with gestational diabetes mellitus. Health Vocational Education, 38, 148–151.

[aphw70127-bib-0055] Liu, X. , Chen, S. , Fei, W. , Wu, Y. , & Cang, X. (2022). Effects of narrative nursing on negative emotions, self‐management ability, and quality of life in adult patients with epilepsy. General Nursing, 20, 526–529.

[aphw70127-bib-0056] Lopez‐Olivo, M. A. , Des Bordes, J. K. , Lin, H. , Volk, R. J. , Rizvi, T. , & Suarez‐Almazor, M. E. (2021). A randomized controlled trial comparing two self‐administered educational strategies for patients with knee osteoarthritis. ACR Open Rheumatology, 3(3), 185–195. 10.1002/acr2.11222 33590950 PMC7966878

[aphw70127-bib-0057] Lopez‐Olivo, M. A. , Lin, H. , Rizvi, T. , Barbo Barthel, A. , Ingleshwar, A. , Des Bordes, J. K. A. , Jibaja‐Weiss, M. , Volk, R. J. , & Suarez‐Almazor, M. E. (2021). Randomized controlled trial of patient education tools for patients with rheumatoid arthritis. Arthritis Care & Research, 73(10), 1470–1478. 10.1002/acr.24362 32583971 PMC10521328

[aphw70127-bib-0058] Lorig, K. R. , & Holman, H. (2003). Self‐management education: History, definition, outcomes, and mechanisms. Annals of Behavioral Medicine: a Publication of the Society of Behavioral Medicine, 26(1), 1–7. 10.1207/S15324796ABM2601_01 12867348

[aphw70127-bib-0059] Loy, M. , & Kowalsky, R. (2024). Narrative medicine: The power of shared stories to enhance inclusive clinical care, clinician well‐being, and medical education. The Permanente Journal, 28(2), 93–101. 10.7812/TPP/23.116 38225914 PMC11232909

[aphw70127-bib-0060] McCaughan, E. , Prue, G. , McSorley, O. , Northouse, L. , Schafenacker, A. , & Parahoo, K. (2013). A randomized controlled trial of a self‐management psychosocial intervention for men with prostate cancer and their partners: A study protocol. Journal of Advanced Nursing, 69(11), 2572–2583. 10.1111/jan.12132 23528148

[aphw70127-bib-0061] Metanmo, S. , Finbråten, H. S. , Bøggild, H. , Nowak, P. , Griebler, R. , Guttersrud, Ø. , Bíró, É. , Unim, B. , Charafeddine, R. , Griese, L. , Kucera, Z. , Le, C. , Schaeffer, D. , Vrdelja, M. , & Mancini, J. (2024). Communicative health literacy and associated variables in nine European countries: Results from the HLS19 survey. Scientific Reports, 14(1), 30245. 10.1038/s41598-024-79327-w 39632907 PMC11618785

[aphw70127-bib-0062] Montalbano, L. , Ferrante, G. , Alesi, M. , & La Grutta, S. (2023). Integrating self‐efficacy in the cyclical process of paediatric asthma management: A new perspective. Psychology, Health & Medicine, 28(6), 1582–1590. 10.1080/13548506.2022.2029918 35073809

[aphw70127-bib-0063] Moreau, K. A. , Eady, K. , Sikora, L. , & Horsley, T. (2018). Digital storytelling in health professions education: A systematic review. BMC Medical Education, 18(1), 208. 10.1186/s12909-018-1320-1 30200945 PMC6131857

[aphw70127-bib-0064] Murphy, S. T. , Frank, L. B. , Chatterjee, J. S. , & Báezconde‐Garbanati, L. (2013). Narrative versus nonnarrative: The role of identification, transportation, and emotion in reducing health disparities. Journal of Communication, 63(1), 116–137. 10.1111/jcom.12007 PMC3857102 24347679

[aphw70127-bib-0065] Mwangi, K. , Gathecha, G. , Nyamongo, M. , Kimaiyo, S. , Kamano, J. , Bukachi, F. , Odhiambo, F. , Meme, H. , Abubakar, H. , & Mwangi, N. (2021). Reframing non‐communicable diseases and injuries for equity in the era of universal health coverage: Findings and recommendations from the Kenya NCDI poverty commission. Annals of Global Health, 87(1), 3. 10.5334/aogh.3085 33505862 PMC7792462

[aphw70127-bib-0066] Naderbagi, A. , Loblay, V. , Zahed, I. U. M. , Ekambareshwar, M. , Poulsen, A. , Song, Y. J. C. , Ospina‐Pinillos, L. , Krausz, M. , Kamel, M. M. , Hickie, I. B. , & LaMonica, H. M. (2024). Cultural and contextual adaptation of digital health interventions: Narrative review. Journal of Medical Internet Research, 26(1), e55130. 10.2196/55130 38980719 PMC11267096

[aphw70127-bib-0067] Oschatz, C. , & Marker, C. (2020). Long‐term persuasive effects in narrative communication research: A meta‐analysis. Journal of Communication, 70(4), 473–496. 10.1093/joc/jqaa017

[aphw70127-bib-0068] Perrier, M.‐J. , & Martin Ginis, K. A. (2017). Narrative interventions for health screening behaviours: A systematic review. Journal of Health Psychology, 22(3), 375–393. 10.1177/1359105315603463 26359288

[aphw70127-bib-0069] Perski, O. , Blandford, A. , West, R. , & Michie, S. (2017). Conceptualising engagement with digital behaviour change interventions: A systematic review using principles from critical interpretive synthesis. Translational Behavioral Medicine, 7(2), 254–267. 10.1007/s13142-016-0453-1 27966189 PMC5526809

[aphw70127-bib-0070] Petty, R. E. , & Cacioppo, J. T. (1986). The elaboration likelihood model of persuasion. In Advances in experimental social psychology (Vol. 19) (pp. 123–205). Elsevier. 10.1016/s0065-2601(08)60214-2

[aphw70127-bib-0071] Pham, T. V. , Koirala, R. , Wainberg, M. L. , & Kohrt, B. A. (2020). Reassessing the mental health treatment gap: What happens if we include the impact of traditional healing on mental illness? Community Mental Health Journal, 57(4), 777–791. 10.1007/s10597-020-00705-5 32894398 PMC7936992

[aphw70127-bib-0072] Ranjit, N. , Menendez, T. , Creamer, M. , Hussaini, A. , Potratz, C. R. , & Hoelscher, D. M. (2015). Narrative communication as a strategy to improve diet and activity in low‐income families: The use of role model stories. American Journal of Health Education, 46(2), 99–108. 10.1080/19325037.2014.999962

[aphw70127-bib-0073] Richardson, J. , Loyola‐Sanchez, A. , Sinclair, S. , Harris, J. , Letts, L. , MacIntyre, N. J. , Wilkins, S. , Burgos‐Martinez, G. , Wishart, L. , McBay, C. , & Martin Ginis, K. (2014). Self‐management interventions for chronic disease: A systematic scoping review. Clinical Rehabilitation, 28(11), 1067–1077. 10.1177/0269215514532478 24784031

[aphw70127-bib-0074] Roikjær, S. G. , Gärtner, H. S. , & Timm, H. (2022). Use of narrative methods in rehabilitation and palliative care in Scandinavian countries: A scoping review. Scandinavian Journal of Caring Sciences, 36(2), 346–381. 10.1111/scs.13050 34882807

[aphw70127-bib-0075] Rokeach, M. (1968). A theory of organization and change within value‐attitude systems1. Journal of Social Issues, 24(1), 13–33. 10.1111/j.1540-4560.1968.tb01466.x

[aphw70127-bib-0076] Rudnicka, E. , Napierała, P. , Podfigurna, A. , Męczekalski, B. , Smolarczyk, R. , & Grymowicz, M. (2020). The World Health Organization (WHO) approach to healthy ageing. Maturitas, 139, 6–11. 10.1016/j.maturitas.2020.05.018 32747042 PMC7250103

[aphw70127-bib-0077] Shaffer, V. A. , Focella, E. S. , Hathaway, A. , Scherer, L. D. , & Zikmund‐Fisher, B. J. (2018). On the usefulness of narratives: An interdisciplinary review and theoretical model. Annals of Behavioral Medicine, 52(5), 429–442 Oxford University Press. 10.1093/abm/kax008 29684135 PMC6369912

[aphw70127-bib-0078] Song, T. , & Cheng, G. (2023). A study on the impact of narrative nursing on negative emotions, self‐management ability and quality of life of adult epilepsy patients. Chinese Journal of Health Care, 41, 133–136.

[aphw70127-bib-0079] Tian, Q. , Feng, T. , Zhang, S. , Wen, J. , & Tang, P. (2024). Effects of five‐step narrative nursing on self‐management ability and negative emotions in patients with diabetes. Journal of Chengde Medical College, 41, 496–499. 10.15921/j.cnki.cyxb.2024.06.003

[aphw70127-bib-0080] Timmermans, L. , Golder, E. , Decat, P. , Foulon, V. , Van Hecke, A. , & Schoenmakers, B. (2023). Characteristics of self‐management support (SMS) interventions and their impact on quality of life (QoL) in adults with chronic diseases: An umbrella review of systematic reviews. Health Policy, 135, 104880. 37536047 10.1016/j.healthpol.2023.104880

[aphw70127-bib-0081] Wang, Y. , Jia, L. , & Liu, Y. (2025). Effects of narrative nursing on emotional state, perceived stress, and self‐efficacy in patients with diabetic nephropathy. Psychological Monthly, 20, 184–186. 10.19738/j.cnki.psy.2025.05.056

[aphw70127-bib-0082] Winterbottom, A. , Bekker, H. , Conner, M. , & Mooney, A. (2008). Does narrative information bias individual's decision making? A systematic review. In Social Science & Medicine (Vol. 67, Issue 12) (pp. 2079–2088). Elsevier BV. 10.1016/j.socscimed.2008.09.037 18951673

[aphw70127-bib-0083] World Health Organization . (2013). Global action plan for the prevention and control of noncommunicable diseases 2013‐2020. World Health Organization.

[aphw70127-bib-0084] World Health Organization . (2024, December 23). Non communicable diseases. https://www.who.int/news-room/fact-sheets/detail/noncommunicable-diseases

[aphw70127-bib-0085] Yang, L. (2022). Effects of narrative nursing on self‐efficacy and resilience in patients undergoing heart valve replacement surgery. Journal of Shanxi Health Vocational College, 32, 94–95.

[aphw70127-bib-0086] Yang, Y. , Xu, J. , Hu, Y. , Hu, J. , & Jiang, A. (2020). The experience of patients with cancer on narrative practice: A systematic review and meta‐synthesis. Health Expectations, 23(2), 274–283. 10.1111/hex.13003 31944492 PMC7104641

[aphw70127-bib-0087] Yıldırım, J. G. (2020). Self‐management of chronic diseases: A descriptive phenomenological study. Social Work in Public Health, 36(2), 300–310. 10.1080/19371918.2020.1859034 33378254

[aphw70127-bib-0088] Zarifsaniey, N. , Shirazi, M. O. , Mehrabi, M. , & Bagheri, Z. (2022). Promoting self‐management behaviors in adolescents with type 1 diabetes, using digital storytelling: A pilot randomized controlled trial. BMC Endocrine Disorders, 22(1), 74. 10.1186/s12902-022-00988-7 35317771 PMC8941790

[aphw70127-bib-0089] Zhang, W. , Liu, Z. , Chen, L. , Hou, D. , & Zhu, S. (2022). Effects of narrative nursing on negative emotions and self‐management ability in patients with tumor PICC catheterization. Hospital Management Forum, 39, 56–60.

[aphw70127-bib-0090] Zheng, L. , Liang, Y. , & Xie, J. (2024, July). The Application Effect of Narrative Nursing Intervention Model in Patients with Artificial Anus and Its Impact on Self‐ management Level and Psychology .

[aphw70127-bib-0091] Zhou, X. , Shao, J. , & Zhao, Y. (2020). Effects of narrative nursing on negative emotions and self‐management behaviors in patients with decompensated liver cirrhosis. Contemporary Nurses (Mid‐Month Edition), 27, 22–24. 10.19792/j.cnki.1006-6411.2020.17.009

[aphw70127-bib-0092] Zhu, D. , Jia, Z. , Li, X. , Yan, L. , Chen, X. , Lian, C. , & Lian, M. (2024). Effects of health management based on narrative medicine model on self‐management cognitive function and limb function in perimenopausal stroke patients. Chinese Journal of Maternal and Child Health, 39, 1883–1886. 10.19829/j.zgfybj.issn.1001-4411.2024.10.036

[aphw70127-bib-0093] Zhu, J. , Chen, S.‐H. , Guo, J.‐Y. , Li, W. , Li, X.‐T. , Huang, L.‐H. , & Ye, M. (2024). Effect of digital storytelling intervention on resilience, self‐efficacy and quality of life among patients with non‐small cell lung cancer (NSCLC): A randomized controlled trial. European Journal of Oncology Nursing, 69, 102535. 10.1016/j.ejon.2024.102535 38401347

