# Supplementary material for: Effects of narrative‐based interventions on self‐efficacy and self‐management in chronic disease: A systematic review and meta‐analysis
Source: Appl Psychol Health Well Being. 2026 Feb 17;18(1):e70127. doi: 10.1111/aphw.70127 (PMC12914092; doi:10.1111/aphw.70127)

**Effects of Narrative-based Interventions on Self-Efficacy and Self-Management in Chronic Disease: A Systematic Review and Meta-Analysis**

**Supplementary Materials**

[**Table S 1:Search strategies** 2](#__RefHeading___Toc215855561)

[**Table S 2: Characteristics of Included Studies** 5](#__RefHeading___Toc215855562)

[**Table S 3:Summary of meta-regression results of self-efficacy.** 19](#__RefHeading___Toc215855563)

[**Table S 4:Summary of meta-regression results of self-management.** 19](#__RefHeading___Toc215855564)

[**Table S 5:Results of leave-one-out method in sensitivity analysis for self-efficacy** 20](#__RefHeading___Toc215855565)

[**Table S 6:Results of leave-one-out method in sensitivity analysis for self-management** 21](#__RefHeading___Toc215855566)

[**Table S 7:An Egger’s Test for Publication Bias in Narrative-based Interventions on Self-Efficacy** 22](#__RefHeading___Toc215855567)

[**Table S 8:An Egger’s Test for Publication Bias in Narrative-based Interventions on Self- Management** 23](#__RefHeading___Toc215855568)

[**Figure S 1:Risk of Bias Assessment of 34 Included Studies** 23](#__RefHeading___Toc215855569)

[**Figure S 2:Plot for Subgroup Analysis Based on Follow-Up Time: Self-Efficacy.** 25](#__RefHeading___Toc215855570)

**Table S 1:Search strategies**

| PubMed | | |
| --- | --- | --- |
|  | (("Narrative Therapy"[MeSH] OR "Narrative Medicine"[MeSH] OR "Narration"[MeSH] OR "Narrative Therapy" OR "Narrative Medicine" OR "Narration*" OR "Personal Narrative" OR "Narrative" OR "Storytelling" OR "Anecdot*" OR "Narrative Counseling" OR "Narrative-Based Therapy" OR "Story-Based Therapy" OR "Life Story Therapy" OR "Narrative Coaching" OR "Storytelling Therapy" OR "Narrating" OR "Description")  AND  ("Self-Management"[MeSH] OR "Self Care"[MeSH] OR "Self Efficacy"[MeSH] OR "Self-Esteem"[MeSH] OR "Self-Confidence"[MeSH] OR "Patient Compliance"[MeSH] OR "Disease Management"[MeSH] OR "Medication Adherence"[MeSH] OR "Self Administration"[MeSH] OR "Self Control"[MeSH] OR "Self Management" OR "Self Care" OR "Self Efficacy" OR "Self-Esteem" OR "Self-Confidence" OR "Patient Compliance" OR "Disease Management" OR "Medication Adherence" OR "Self Administration" OR "Self Control"))  AND  ("Randomized Controlled Trial"[Publication Type] OR "RCT" OR "Randomized Trial") | 416 |
| Embase | | |
|  | (exp 'narrative therapy'/ or exp 'narration'/ or (narrative therap* or narrative medicine or narration* or personal narrative or narrative or storytelling or anecdot* or narrative counseling or narrative-based therap* or story-based therap* or life story therap* or narrative coaching or storytelling therap* or narrating or description).ti,ab,kw.) and (exp 'self care'/ or exp 'self concept'/ or exp 'self efficacy'/ or exp 'self esteem'/ or exp 'self confidence'/ or exp 'treatment compliance'/ or exp 'disease management'/ or exp 'medication adherence'/ or exp 'self medication'/ or exp 'self control'/ or (self-management or self-care or self-efficacy or self-esteem or self-confidence or patient compliance or disease management or medication adherence or self administration or self control).ti,ab,kw.) and (exp 'randomized controlled trial'/ or (RCT or randomized controlled trial or randomized trial).ti,ab,kw.) | 3974 |
| PsycINFO | | |
|  | [STRICT] (MH "Narrative Therapy" OR MH "Narrative Medicine" OR MH "Narration" OR "narrative therap*" OR "narrative medicine" OR "narration*" OR "personal narrative" OR "narrative" OR "storytelling" OR "anecdot*" OR "narrative counseling" OR "narrative-based therap*" OR "story-based therap*" OR "life story therap*" OR "narrative coaching" OR "storytelling therap*" OR "narrating" OR "description")  AND  (MH "Self-Management" OR MH "Self Care" OR MH "Self Efficacy" OR MH "Self-Esteem" OR MH "Self-Confidence" OR MH "Patient Compliance" OR MH "Disease Management" OR MH "Medication Adherence" OR MH "Self Administration" OR MH "Self Control" OR "self management" OR "self care" OR "self efficacy" OR "self-esteem" OR "self-confidence" OR "patient compliance" OR "disease management" OR "medication adherence" OR "self administration" OR "self control")  AND  (MH "Randomized Controlled Trials" OR "randomized controlled trial" OR "RCT" OR "randomized trial") | 94 |
| CNKI | | |
|  | (篇关摘: 叙事治疗 + 叙事医学 + 叙述 + 个人叙事 + 叙事 + 讲故事 + 轶事 + 故事治疗 + 叙事指导 + 讲故事治疗 + 叙述) AND (篇关摘: 自我管理 + 自我护理 + 自我效能 + 自尊 + 自信 + 患者依从性 + 疾病管理 + 用药依从性 + 自我控制) AND (篇关摘: 随机对照试验 + RCT + 随机试验) | 494 |
| CINAHL Plus | | |
|  | (MH "Narrative Therapy" OR MH "Narrative Medicine" OR MH "Narration" OR TI "Narrative Therapy" OR AB "Narrative Therapy" OR TI "Narrative Medicine" OR AB "Narrative Medicine" OR TI "Narration*" OR AB "Narration*" OR TI "Personal Narrative" OR AB "Personal Narrative" OR TI "Narrative" OR AB "Narrative" OR TI "Storytelling" OR AB "Storytelling" OR TI "Anecdot*" OR AB "Anecdot*" OR TI "Narrative Counseling" OR AB "Narrative Counseling" OR TI "Narrative-Based Therapy" OR AB "Narrative-Based Therapy" OR TI "Story-Based Therapy" OR AB "Story-Based Therapy" OR TI "Life Story Therapy" OR AB "Life Story Therapy" OR TI "Narrative Coaching" OR AB "Narrative Coaching" OR TI "Storytelling Therapy" OR AB "Storytelling Therapy" OR TI "Narrating" OR AB "Narrating" OR TI "Description" OR AB "Description")  AND  (MH "Self-Management" OR MH "Self Care" OR MH "Self Efficacy" OR MH "Self-Esteem" OR MH "Self-Confidence" OR MH "Patient Compliance" OR MH "Disease Management" OR MH "Medication Adherence" OR MH "Self Administration" OR MH "Self Control" OR TI "Self-Management" OR AB "Self-Management" OR TI "Self Care" OR AB "Self Care" OR TI "Self Efficacy" OR AB "Self Efficacy" OR TI "Self-Esteem" OR AB "Self-Esteem" OR TI "Self-Confidence" OR AB "Self-Confidence" OR TI "Patient Compliance" OR AB "Patient Compliance" OR TI "Disease Management" OR AB "Disease Management" OR TI "Medication Adherence" OR AB "Medication Adherence" OR TI "Self Administration" OR AB "Self Administration" OR TI "Self Control" OR AB "Self Control")  AND  (MH "Randomized Controlled Trials" OR TI "Randomized Controlled Trial" OR AB "Randomized Controlled Trial" OR TI "RCT" OR AB "RCT" OR TI "Randomized Controlled Trials" OR AB "Randomized Controlled Trials" OR TI "randomized" OR AB "randomized" OR TI "randomised" OR AB "randomised" OR TI "controlled clinical trial" OR AB "controlled clinical trial" OR TI "randomly assigned" OR AB "randomly assigned" OR TI "clinical trial" OR AB "clinical trial") | 115 |
|  | Cochrane library |  |
| #1 | MeSH descriptor: [Narrative Medicine] explode all trees | 16 |
| #2 | MeSH descriptor: [Narrative Therapy] explode all trees | 64 |
| #3 | MeSH descriptor: [Narration] explode all trees | 356 |
| #4 | MeSH descriptor: [Anecdotes] explode all trees | 0 |
| #5 | ((narrative) OR (storytelling) OR (Anecdote) OR (Narrative counseling) OR (Narrative-based therapy) OR (Story-based therapy) OR (Life story therapy) OR (Narrative coaching) OR (Storytelling therapy) OR (Narrating) OR (Description)):ti,ab,kw | 12441 |
| #6 | #1 OR #2 OR #3 OR #4 OR #5 | 12516 |
| #7 | MeSH descriptor: [Self-Management] explode all trees | 1455 |
| #8 | MeSH descriptor: [Self Care] explode all trees | 7939 |
| #9 | MeSH descriptor: [Self Efficacy] explode all trees | 4729 |
| #10 | MeSH descriptor: [Self-Control] explode all trees | 860 |
| #11 | MeSH descriptor: [Patient Compliance] explode all trees | 20456 |
| #12 | MeSH descriptor: [Disease Management] explode all trees | 7776 |
| #13 | MeSH descriptor: [Medication Adherence] explode all trees | 9137 |
| #14 | MeSH descriptor: [Self Administration] explode all trees | 933 |
| #15 | #7 OR #8 OR #9 OR #10 OR #11 OR #12 OR #13 OR #14 | 39905 |
| #16 | #6 AND #15 | 317 |

**Table S 2: Characteristics of Included Studies**

| **Author(s)** | **Location/(year)** | **Study design** | **Population** | **Sample size N (I/C)** | **Mean age (SD)** | **Intervention** | **Control** | **Intensity and duration** | **Time point of measures** | **Assessment tools** |
| --- | --- | --- | --- | --- | --- | --- | --- | --- | --- | --- |
| Andreae et al. | USA (2021) | Cluster randomized controlled trial | Type 2 Diabetes | 203/270 (IA: 165/239) | 57.16 (10.95) | 11-session telephonic peer coaching + storytelling DVDs focusing on medication adherence | Self-paced general health education program (DVDs on unrelated topics e.g., cancer, osteoporosis) + brief check-in calls | 11 sessions over 6 months (6-wk intensive phase + maintenance) | Baseline T0, T1: 6 months | SEAMS; PDSMS |
| Appalasamy, J., et al. | Malaysia (2020a) | Single-blind RCT | Stroke | 108/108 (IA: 89 / 88，85/82) | 55.5 (12.3) | Standard care + face-to-face video narratives | Standard care only (pamphlets, teach-back method) | 3 sessions (Baseline T0, 3 months T1, 6 months T2); over 6 months | Baseline T0,  6 months T2 12 months T3 | MUSE |
| Appalasamy, Q., et al. | Malaysia (2020b) | Feasibility RCT | Post-stroke patients | 30/30  (IA：27/27) | 54.5 (12.4) | Standard care + face-to-face video narratives | Standard care only (pamphlets, teach-back method) | Single session of video viewing (approx. 10 mins) at baseline. | Baseline T0,  Baseline T0, | MUSE |
| Barroso et al. | USA (2008) | RCT | HIV-infected women, IHSS score ≥40 | 51/49  (IA: 51/49) | 45.9 (9.7) | iPod Touch with 45-min narrative video + standard care | iPod Touch (no video) + standard care | Watch video ≥1x/week for first 4 weeks | Baseline T0,  30 days T1,  90 days T2 | CSES |
| Bell et al. | USA (2021) | RCT | Type 1 Diabetes | 94/97  (IA: 94/97) | 14.62 (1.55) | 3 text-based narrative messages (avg 450 words) about T1D management. | Standard of care messages | Single exposure (≥30 sec per message) | Baseline T0, Immediate post-exposure T1. | Self-efficacy: A 6-item modified of the Self-efficacy for Diabetes Management scale |
| Campbell et al. | Australia (2013) | RCT | Type 2 Diabetes | 335/335 (IA: 284/312) | 55.73 (8.71) | Standard brochures + a DVD with patient narratives and a workbook. | Standard brochures only. | 3-week DVD viewing (1 module/week). | Baseline T0,  4 weeks T1 | A/E DMSES |
| Corgan et al. | USA (2008) | Pilot RCT | Cancer | 5/5  (IA: 3/4) | Range: 48-74 years | Nuse-led storytelling | Usual care | 12 weeks, 1.5 hours/session. | Baseline T0, 3 months T1 | PSES |
| Cui et al. | China (2025) | RCT | Cancer | 46/46  (IA: 46/46) | 43.5 (7.2) | Cox concept-based phased narrative therapy involving problem externalization, structured hope-finding interviews, strength identification, and resource linkage | Usual care | Hospitalization period | Baseline T0, Immediate post-exposure T1. | SMBS |
| Dennick et al. | UK (2015) | Feasibility RCT | Type 2 Diabetes | 41  (IA:23/18) | 65.6 (9.9) | Written Emotional Disclosure (WED): write about stressful experiences for 20 min × 3 days | Neutral Writing: describe previous day’s activities | 20 min/session, 3 sessions over 1 week | Baseline T0,  3 months T1 | Diabetes Self-care Activities questionnaire |
| Falzon et al. | France (2015) | RCT | Breast Cancer | 56/52/50 (IA：56/52/50) | 54.38 (13.0) | Narrative: Testimony of a physically active survivor (Testimony Group) | 1. Informational: Expert recommendations (Recommendations Group). 2. No message (Control Group.). | Single exposure (reading a message). | Baseline T0, Immediate post-exposure T1. | Exercise Self-Efficacy |
| Feng, B. et al. | USA (2021) | RCT | Chronic pain on opioids | 183/182 (IA: 128 / 111) | 37 (11.8) | Narrative-based video | CDC informational pamphlet | Video: ≥30 sec; Pamphlet: ≥5 sec | Baseline T0, Immediate post-exposure T1. | Self-efficacy for tapering |
| Feng, X. et al. | China (2021) | RCT | Patients with advanced pancreatitis and diabetes | 24/24  (IA: 24/24) | 50.61(4.07) | Narrative Nursing Model + Routine Care | Usual care | 3 times/week, 20-30 min each, for 4 weeks | Baseline T0, 1 months T1. | ESCA |
| Gao and Yang | China (2022) | RCT | Cancer | 43/43  (IA: 43/43) | 61.3 (4.5) | Narrative medicine-guided psychological intervention involving structured storytelling, co-creation of care plans, and targeted support for both patients and caregivers to reframe the illness experience. | Usual care | 1 month (specific frequency not detailed) | Baseline T0, 1 months T1. | SUPPH |
| Giesler et al. | Germany (2017) | RCT | Cancer | 103/109 (IA: 103/ 109) | 54.1 (11.1) | Access to DIPEx colorectal cancer experience website | Waiting list control group | 2 weeks of access; Median use: 42.21 min | Baseline T0,  2 weeks T1,  6 weeks T2 | CBI-B-D |
| He et al. | China (2020) | RCT | COPD | 30/30  (IA: 30/30) | 75.5 (5.0) | Personalized Narrative Nursing （Including steps such as guiding the narrative, analyzing narrative content, providing positive feedback, and conducting in-depth intervention） | Usual care | 2-3 sessions/week, 20-30 min/session, for 1 month | Baseline T0, 1 months T1. | Self-Efficacy Scale (From daily life, health behaviors, drug treatment, and compliance with medical advice) |
| Huang et al. | China (2024) | RCT | Coronary heart disease | 58/58  (IA: 58/58) | 51.5 (8.2) | COM-B model-based modified narrative care involving co-created thematic videos. | Usual care | 3 months (included video sharing, phone follow-up) | Baseline T0, 3 months T1 | GSES |
| Iannello et al. | Italy (2018) | RCT | Hip Fracture | 21/21  (IA:21/21) | 79.67 (9.03) | Self-Narration Journey (SNJ) | Usual care | 3-5 sessions over 2 weeks, 15-20 min each | Baseline T0, Discharge (Two weeks) T1 | GSES |
| Lee et al. | Canada (2006) | RCT | Breast Cancer, Colorectal Cancer | 41/41  (IA: 35/39) | 56.65 (10.0) | Structured Narrative Meaning-Making Intervention (nurse-led) | Usual care | Up to 4 individualized sessions (up to 120 min each), time from start to end: 32 days. | Baseline T0,  post-exposure T1 | GSES |
| Lely et al. | Netherlands (2022) | RCT | PTSD | 18/15  (IA: 14/12) | 63.81 (6.8) | Narrative Exposure Therapy | Present-Centered Therapy | 11 sessions, frequency 1-2 weeks per session (adapted to preference), average treatment duration ~22 weeks | Baseline T0,  post-exposure T1, 4 months T2 | GSES |
| Liu, S. et al. | China (2020) | RCT | Gestational Diabetes | 30/30 (IA:30/30) | 18-45 | Narrative nursing | Usual care | 3-4 sessions, 35-50 min/session. | Baseline T0, 1 months T1. | 2- DSCS |
| Liu, X. et al. | China (2022) | RCT | Epilepsy | 36/36  (IA: 36/36) | 41.8 (7.3) | Narrative nursing (involving structured attention, understanding, response (problem externalization, solution, rewriting, witnessing), and reflection phases to reframe the illness experience) | Usual care | Inpatient: 2 sessions; Discharge: 1 session; Post-discharge: Biweekly for 1 month, then monthly for 6 months (45 min/session) | Baseline T0, 6 months T1 | ESMS |
| Lopez-Olivo et al. | USA (2021b) | RCT | Rheumatoid Arthritis | 111 / 110 (IA: 47/58, 42/35) | 50.8 (13.3) | Video (3-7min) + written booklet | Booklet alone. | Single exposure. Video length: 20 mins. Booklet review: self-paced. Total time for combo group: 25-45 mins; | Baseline T0, 3 months T1,  6 months T2 | ASES |
| Lopez-Olivo et al. | USA (2021a) | RCT | Knee Osteoarthritis | 109/110 (IA: 83/90, 98/91) | 64.6 (8.3) | Video (3-7min) + written booklet | Booklet alone. | Single exposure. Video: series of dramatized episodes (total length not specified, episodes 3-7 mins each). | Baseline T0, 3 months T1,  6 months T2 | ASES |
| McCauley et al. | USA (2011) | RCT | Chronic illnesses | 51/49  (IA: 51/49) | 65.8 (9.6) | Video & workbook featuring patient narratives on using spiritual beliefs to cope with illness. | Educational video & workbook on cardiac risk reduction | 28-min video + 4-week workbook | Baseline To,  5–8 weeks post-exposure T1 | Self-efficacy scale |
| Song and Cheng | China (2023) | RCT | Epilepsy | 50/50  (IA: 50/50) | 44.9 (5.8) | Narrative nursing" involved forming a specialized team, active listening, encouraging patients to tell their stories, helping them find meaning and positive (power/force), and providing targeted guidance based on their narratives, | Usual care | Hospitalization | Baseline To, post-exposure T1 | ESMS |
| Tian et al. | China (2024) | RCT | Diabetes | 36/36  (IA: 36/36) | 65.2 (6.3) | Narrative nursing involving structured listening, absorbing, understanding, responding, and retelling to reframe the illness experience and co-create personalized management plans | Usual care | 3 months (specific frequency not detailed) | Baseline T0, 3 months T1 | SDSCA |
| Wang et al. | China (2025) | RCT | Diabetic nephropathy | 60/60  (IA: 60/60) | 54.04 (5.39) | Narrative nursing (externalization, deconstruction, rewriting, external witnesses, and therapeutic documents) | Usual care | 8 weeks. Specific frequency not detailed. | Baseline T0, 2 months T1 | GSES |
| Yang | China (2022) | RCT | Undergoing heart valve replacement | 45/45  (IA: 45/45) | 43.73 (5.52) | Narrative Nursing (3 steps: Externalization, Deconstruction, Rewriting) | Usual care | Individual sessions, from admission until discharge (Duration varied per patient's hospital stay) | Baseline To, post-exposure (discharge) T1 | GSES |
| Zarifsaniey et al. | Iran (2022) | Pilot RCT | Type 1 diabetes | 33/33  (IA: 33/33) | 12.54 (3.46) | Digital storytelling | Wait-list | 35-min animation; 10-min biweekly phone calls; 3 months | Baseline T0,  3 months T1 | SMOD-A |
| Zhang. et al | China (2022) | RCT | Cancer | 40/40  (IA: 40/40) | 65.23 (8.38) | Narrative Nursing (Individual sessions using externalization, deconstruction, rewriting techniques) | Usual care | Individual sessions, 15-30 mins, during weekly clinic visits, for 1 month | Baseline T0, 1 months T1. | CPPSM |
| Zheng et al. | China (2024) | RCT | Cancer | 30/30 (IA:30/30) | 51.6 (6.66) | Narrative nursing-based intervention (involving story listening, content analysis, cognitive reframing, and personalized stoma care education) | Usual care | 3 months (specific frequency not detailed) | Baseline T0, 3 months T1 | Self-management Scale: 5 domains |
| Zhou et al. | China (2020) | RCT | Decompensated cirrhosis | 60/60  (IA: 60/60) | 50.78 (6.81) | Narrative nursing (building trust, listening to narratives, externalizing problems, deconstructing and rewriting stories) | Usual care | Twice/week, 20 min/session. Duration: 3 months. | Baseline T0, 3 months T1 | Self-management Scale for Cirrhosis |
| Zhu, J., et al. | China (2024) | RCT | Non-Small Cell Lung Cancer (NSCLC) | 45 / 45 (IA: 44/43,43/43,42/42) | 52.27 (9.6) | Digital storytelling intervention | Usual care | 4 weekly videos (15 min each), total 4 weeks | Baseline T0,  post-inter T1,  1-month T2, 3months T3 | SUPPH |
| Zhu, D., et al. | China (2024) | RCT | Stroke | 50/50  (IA: 50/50) | 49.16 (4.54) | Narrative medicine-based health management involving empathetic listening, co-creation of personalized plans, and guided storytelling to reframe the illness experience. | Usual care | 6 months (specific frequency not detailed) | Baseline T0, 6 months T1 | Self-health Management Scale |
| RCT: Randomized Controlled Trial, T1D: Type 1 Diabetes, T2D: Type 2 Diabetes, **COPD:** Chronic Obstructive Pulmonary Disease, **HIV:** Human Immunodeficiency Virus, **PTSD:** Post-Traumatic Stress Disorder, **COM-B:** Capability, Opportunity, Motivation – Behavior, SEAMS: Self-Efficacy for Appropriate Medication Use Scale, PDSMS: Perceived Diabetes Self-Management Scale, MUSE: Medication Understanding and Use Self-Efficacy Scale, CSES: Coping Self-Efficacy Scale, A or E DMSES: Attitude or Efficacy Diabetes Management Self-Efficacy Scale, PSES: Psychological Self-Efficacy Scale, SMBS: Self-Management Behavior Scale, ESCA: Exercise Self-Care Agency Scale, SUPPH: Strategies Used by People to Promote Health, CBI: Cancer Behavior Inventory, GSES: General Self-Efficacy Scale, 2-DSCS: 2-Dimensional Self-Care Scale, ESMS: Epilepsy Self-Management Scale, ASES: Arthritis Self-Efficacy Scale, SMOD-A: Self-Management of Type 1 Diabetes for Adolescents Scale, CPPSM: Cancer Patient PICC Self-Management Scale, SDSCA: Summary of Diabetes Self-Care Activities. SD: Standard Deviation. N: Total Sample Size. IA: Intention-to-Analysis. I/C: Intervention / Control. PICC: Peripherally Inserted Central Catheter. | | | | | | | | | | |

**Table S 3:Summary of meta-regression results of self-efficacy.**

| **Variable** | **Coefficient** | **SE.** | **t** | **P>|t|** |
| --- | --- | --- | --- | --- |
| Income-level | 0.8416587 | 0.4011756 | 2.1 | 0.055 |
| Self-efficacy type | -0.0862676 | 0.313002 | -0.28 | 0.787 |
| Age | -0.1572052 | 0.2187897 | -0.72 | 0.484 |
| Disease-type | -0.0987428 | 0.1132969 | -0.87 | 0.398 |
| Intervention-type | -0.0365009 | 0.2826141 | -0.13 | 0.899 |
| Duration | -0.1988744 | 0.3047172 | -0.65 | 0.525 |
| Publish period | 0.3450649 | 0.4062136 | 0.85 | 0.41 |
| _cons | 0.1608217 | 1.483155 | 0.11 | 0.915 |

**Table S 4:Summary of meta-regression results of self-management.**

| **Variable** | **Coefficient** | **SE.** | **t** | **P>|t|** |
| --- | --- | --- | --- | --- |
| Age | 0.17 | 0.82 | 0.21 | 0.842 |
| Disease-type | -2.048 | 1.404 | -1.46 | 0.195 |
| Intervention-type | -2.713 | 1.478 | -1.84 | 0.116 |
| Duration | 2.224 | 1.524 | 1.46 | 0.195 |
| Publish period | 1.799 | 1.45 | 1.24 | 0.261 |
| _cons | 1.606 | 3.058 | 0.53 | 0.618 |

**Table S 5:Results of leave-one-out method in sensitivity analysis for self-efficacy**

| **Study (Year)** | **Estimate [95% Conf. Interval]** |
| --- | --- |
| Andreae et al. (2021) | 0.69 [0.37, 1.01] |
| Appalasamy, J. (2020) | 0.65 [0.33, 0.97] |
| Appalasanny, Q. (2020a) | 0.66 [0.34, 0.97] |
| Barroso et al. (2014) | 0.68 [0.36, 0.99] |
| Bell et al. (2021) | 0.69 [0.37, 1.01] |
| Campbell et al (2015) | 0.68 [0.35, 1.02] |
| Corgan et al. (2008) | 0.67 [0.37, 0.98] |
| Falzon et al. (2015) | 0.64 [0.32, 0.95] |
| Feng et al. (2021) | 0.67 [0.35, 1.00] |
| Gao and Liu (2022) | 0.68 [0.36, 0.99] |
| Giesler et al. (2017) | 0.55 [0.31, 0.79] |
| He et al. (2020) | 0.65 [0.34, 0.96] |
| Huang et al. (2024) | 0.64 [0.33, 0.96] |
| Iannello et al. (2018) | 0.64 [0.32, 0.95] |
| Lee et al. (2006) | 0.67 [0.35, 0.98] |
| Lely et al. (2022) | 0.71 [0.41, 1.02] |
| Lopez et al. (2021a) | 0.69 [0.38, 1.01] |
| Lopez et al. (2021) | 0.69 [0.37, 1.01] |
| Maccauley et al. (2011) | 0.68 [0.37, 1.00] |
| Wang et al. (2025) | 0.57 [0.29, 0.86] |
| Yang (2022) | 0.62 [0.32, 0.93] |
| Zhu et al. (2024) | 0.65 [0.33, 0.96] |
| Combined | 0.66 [0.35, 0.96] |

**Table S 6:Results of leave-one-out method in sensitivity analysis for self-management**

| **Study (Year)** | **Estimate [95% Conf. Interval]** |
| --- | --- |
| Dennick et al. (2015) | 1.91 [1.19, 2.62] |
| Feng et al. (2021) | 1.75 [1.01, 2.50] |
| Liu, S., et al. (2020) | 1.79 [1.03, 2.55] |
| Liu, X., et al. (2022) | 1.33 [0.75, 1.91] |
| Song and Cheng (2023) | 1.77 [1.00, 2.55] |
| Tian et al. (2024) | 1.84 [1.07, 2.60] |
| Zarifsaniey et al. (2022) | 1.87 [1.13, 2.61] |
| Zhang et al. (2022) | 1.43 [0.82, 2.03] |
| Zheng et al. (2024) | 1.76 [1.00, 2.51] |
| Zhou et al. (2020) | 1.87 [1.11, 2.62] |
| Zhu et al. (2024) | 1.75 [0.98, 2.51] |
| Combined | 1.73 [1.03, 2.42] |

**Table S 7:An Egger’s Test for Publication Bias in Narrative-based Interventions on Self-Efficacy**

| Std_Eff | Coefficient | SE | t | P>|t| | [95% CI] |
| --- | --- | --- | --- | --- | --- |
| slope | -.0990448 | .3422828 | -0.29 | 0.775 | -.8130343 .6149447 |
| bias | 3.592214 | 1.959601 | 1.83 | 0.082 | -.4954415 7.679871 |

CI = confidence interval; SE = standard error **p* < .001.

**Table S 8:An Egger’s Test for Publication Bias in Narrative-based Interventions on Self- Management**

| Std_Eff | Coefficient | SE | *t* | P>|t| | [95% CI] |
| --- | --- | --- | --- | --- | --- |
| slope | -1.889662 | .9330125 | -2.03 | 0.070 | -3.968543 .1892193 |
| bias | 11.95695 | 3.49635 | 3.42 | 0.007 | 4.1666  19.74731 |

CI = confidence interval; SE = standard error **p* < .001.

**Figure S 1:Risk of Bias Assessment of 34 Included Studies**


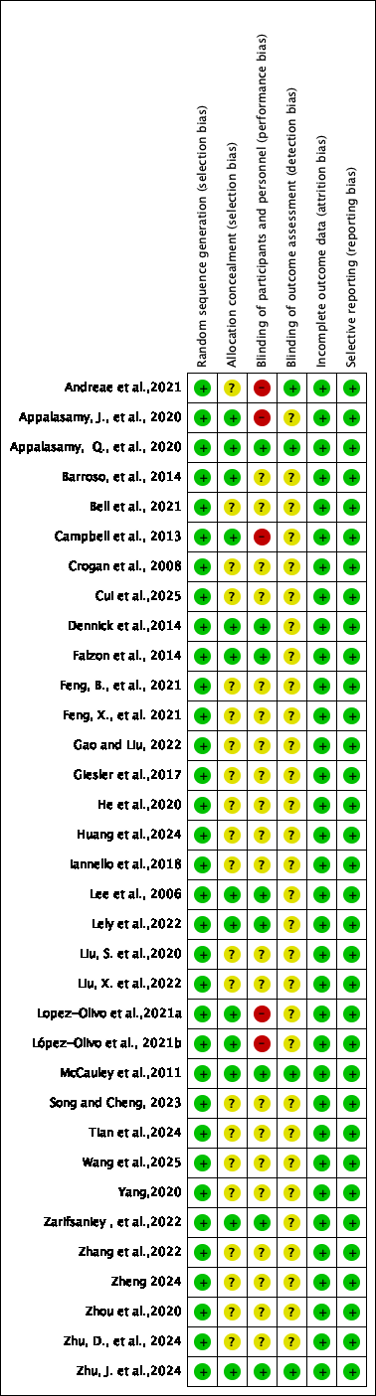


**Figure S 2:Plot for Subgroup Analysis Based on Follow-Up Time: Self-Efficacy.**


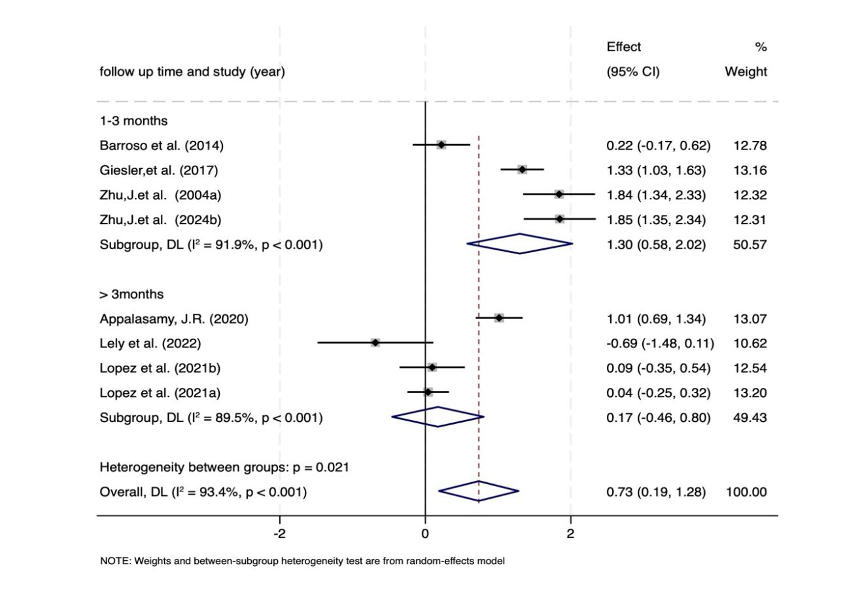

Supplement: Supplementary file 1 — Table S1. Search strategies. Table S2. Characteristics of Included Studies. Table S3. Summary of meta‐regression results. Table S4. Results of leave‐one‐out method in sensitivity analysis. Table S5. Results of leave‐one‐out method in sensitivity analysis. Figure S1. Risk of Bias Assessment of 34 Included Studies. Figure S2. Egger's Test for Publication Bias in a Meta‐Analysis for Self‐Efficacy. Figure S3. Egger's Test for Publication Bias in a Meta‐Analysis for Self‐Management. [file APHW-18-0-s001.doc]
